# Handheld Near-Infrared Spectroscopy-Based Intelligent Screening of Allergen-Containing Flour Cross-Contamination in Gluten-Free Flour

**DOI:** 10.3390/foods15193434

**Published:** 2026-09-25

**Authors:** Siyu Yao, Tong Yu, Zhuoyue Feng, Zulipikaer Rouzi, Xiyu He, Kaiyan Chen

**Affiliations:** Key Laboratory of Environmental Medicine and Engineering, Ministry of Education, and Department of Nutrition and Food Hygiene, School of Public Health, Southeast University, Nanjing 210009, China; 220244305@seu.edu.cn (T.Y.); 213210520@seu.edu.cn (Z.F.); za715524@163.com (Z.R.); u3643918@connect.hku.hk (X.H.); 220254255@seu.edu.cn (K.C.)

**Keywords:** gluten-free flour, allergen-containing flour cross-contamination, handheld NIR spectroscopy, pattern recognition analysis

## Abstract

Controlling cross-contamination with allergen-containing ingredients is critical for ensuring product integrity and safety. Gluten-free flour is particularly susceptible to cross-contamination by common allergen-containing flours such as wheat, peanut, soybean, and sesame during production and processing. This study aims to develop a rapid screening and quantification strategy for allergen-containing flour cross-contamination in gluten-free flour using handheld near-infrared spectroscopy integrated with multivariate data analysis. A total of 330 gluten-free flour samples were prepared, including non-contaminated controls and allergen-containing flour cross-contaminated samples (1–25%, *w*/*w*). Spectral data were collected using a handheld NIR spectrometer. PCA was applied for exploratory data analysis and visualization of sample distribution. SIMCA was used for qualitative classification of cross-contamination presence, followed by PLSR for quantitative prediction of individual allergen-containing flour addition levels. Independent hold-out validation demonstrated 100% accuracy, sensitivity, and specificity for the classification model under the investigation conditions. For quantitative analysis, the PLSR models achieved Rval > 0.96 with low RMSEP values. Overall, the results demonstrate reliable performance and effective discrimination capability under the investigated conditions. This handheld NIR approach would be a rapid, non-destructive screening tool for high-level allergen-containing flour cross-contamination, such as preliminary raw-material screening, in-process monitoring, or rapid screening following potential cross-contact events in gluten-free production.

## 1. Introduction

Food allergy is a major global public health concern [1]. It is defined as an abnormal immune response triggered by the ingestion or exposure to food allergens, typically affecting the skin, gastrointestinal tract, and respiratory system, and in severe cases leading to anaphylactic shock or death [2]. According to the World Health Organization (WHO), approximately 22–25% of the global population suffers from allergic diseases, of which food-induced allergies account for about 77%, highlighting food as the primary trigger of allergic reactions [3]. The prevalence of food allergy continues to rise, and it has been recognized as the sixth major chronic disease worldwide. This increasing burden has been further exacerbated in recent years by changes in dietary patterns and the growing complexity of food processing, both of which have contributed to a higher incidence of food allergy events, posing significant risks to consumer health and substantial challenges to food safety management systems [4]. The Food and Agriculture Organization (FAO) and WHO have identified eight major allergenic food groups, including crustaceans, fish, eggs, milk, peanuts, tree nuts, soybeans, and wheat, which together account for more than 90% of reported food allergic reactions. Among these, peanut, soybean, and wheat are particularly prominent due to their widespread consumption and high allergenic potential, while sesame has also received increasing global attention after being designated as the ninth major allergen in several countries [5]. However, no definitive cure for food allergies currently exists, and strict avoidance of allergen exposure remains the most effective preventive strategy.

With the diversification of modern food systems, allergen avoidance has become increasingly challenging. Gluten-free foods, originally developed to meet the dietary needs of individuals with wheat allergies and celiac disease, are typically produced using naturally gluten-free raw materials. Among these, rice flour is one of the most widely used ingredients due to its favorable sensory properties, nutritional value, and broad availability [6]. Driven by growing health awareness and the rising prevalence of food allergies, global consumption of gluten-free products has been growing at an estimated annual rate of 24%, positioning this sector as an important area of food innovation [7]. However, gluten-free flour is highly vulnerable to cross-contamination during production, processing, transportation, and storage. Wheat is the primary source of gluten contamination, while other allergens such as peanut, soybean, and sesame may also be introduced through shared equipment, raw material mixing, and industrial processing environments. These contaminants are often invisible and difficult to detect visually, yet unintended incorporation of allergen-containing ingredients may pose severe risks to sensitive individuals [8]. Therefore, reliable detection and estimation of allergen-containing ingredient incorporation levels are important for preliminary screening and management of relatively high-level cross-contamination in gluten-free supply chains, particularly during quality control and ingredient handling.

Currently, allergen detection methods mainly include immunoassays (e.g., ELISA, lateral flow immunoassays), chromatographic techniques (LC–MS/MS), and molecular biological methods such as polymerase chain reaction (PCR) [9]. Although these laboratory-based methods are highly sensitive and accurate, they are generally time-consuming, expensive, require skilled operators, and are not well suited for rapid, on-site screening of relatively high-level cross-contamination [10]. In addition, the complexity and diversity of allergenic proteins and matrix interferences make comprehensive detection using a single laboratory method challenging. These limitations highlight the need for rapid, accurate and non-destructive analytical technologies for preliminary monitoring of allergen-containing ingredient cross-contamination. In this context, NIR spectroscopy has emerged as a promising analytical tool for cross-contamination screening because of its rapid analysis, non-destructive nature, minimal sample preparation requirements, and potential for integrated assessment of complex food attributes [11,12]. NIR spectroscopy measures molecular overtone and combination vibrational bands associated with functional groups containing C, N, O, and S atoms, providing rich chemical information related to food composition and structure. However, due to weak, broad, and highly overlapping spectral features, advanced data analysis methods are required for effective interpretation [13].

Multivariate data analysis provides a well-established chemometric framework for interpreting high-dimensional and collinear NIR spectral data. Through techniques such as principal component analysis, classification modeling, and partial least squares regression, multivariate data analysis enables effective extraction of latent information embedded in complex spectral signals and supports both qualitative discrimination and quantitative prediction tasks [14]. The integration of NIR spectroscopy with multivariate data analysis has therefore emerged as a promising approach, enabling rapid and non-destructive screening of compositional differences associated with allergen-containing ingredient contamination in complex food matrices. Driven by advances in optoelectronics and micro-electro-mechanical systems (MEMS), portable (miniaturized) near-infrared spectrometers have become increasingly available in recent decades, enabling in-field and on-site analytical applications [15]. These compact NIR systems eliminate the need for reagents and extensive sample preparation, thereby aligning with the principles of green, low-carbon, and efficient analytical technologies. As a result, portable NIR sensing platforms have shown strong potential for industrial and commercial deployment in food quality monitoring [16].

In recent years, NIR spectroscopy combined with multivariate data analysis has been increasingly explored for allergen related screening in food matrices. Existing studies have demonstrated its potential for identifying individual allergen-containing ingredients or distinguishing contaminated and uncontaminated samples in food matrices such as wheat flour, milk powders, and processed foods [11,12,17]. However, most existing studies have focused on detecting the presence of a specific allergen or performing simplified binary classification between contaminated and non-contaminated samples, which may not fully address the practical need to discriminate among different allergen-containing ingredients involved in food processing cross-contact events. In addition, gluten-free flour represents a particularly challenging matrix for allergen screening because unintended contamination from various allergen sources may occur during processing, storage, or transportation. Despite its practical importance, systematic evaluation of flour cross-contamination involving different allergen-containing ingredients under well-defined individual contamination scenarios in gluten-free matrices remains limited. Moreover, most reported studies rely on benchtop spectroscopic instruments, restricting their potential for rapid, on-site screening applications. Therefore, portable sensing strategies capable of discriminating among different sources of allergen-containing ingredient cross-contamination and estimating their corresponding contamination levels in flour are valuable for rapid preliminary screening and cross-contamination management.

This study aims to develop a rapid (10 s) and on-site screening method for integrated identification and quantification of allergen-containing flour cross-contamination (1% to 25%, w/w) in gluten-free flour using handheld NIR spectroscopy combined with multivariate data analysis. Four allergen-containing flour contamination scenarios, including wheat, peanut, soybean, and sesame, were individually evaluated in rice-based gluten-free flour and detected and quantified in real time. The proposed approach provides an analytical framework for rapid preliminary screening of relatively high-level cross-contamination in complex food matrices under the investigated experimental conditions, with potential applications in ingredient quality control and cross-contamination management.

## 2. Materials and Methods

### 2.1. Sample Preparation

Rice, wheat, peanut, soybean, and sesame were selected as experimental materials. Rice flour was used as the gluten-free matrix due to its gluten-free nature, stable nutritional profile, wide availability, and minimal spectral interference with allergen-containing flour addition signatures. Wheat, peanut, soybean, and sesame were selected as allergen-containing flours recognized worldwide, which may contribute to unintended ingredient cross-contamination during production, processing, transportation, and storage of gluten-free products. All raw materials were purchased from local grocery stores in Nanjing, China and were of food-grade quality, exhibiting normal appearance and color with no signs of mold, insect damage, or off-odor. The corresponding manufacturers/suppliers indicated on the product packaging were Jiangsu Xuyi Tailiang Rice Industry Co., Ltd. (Xuyi, China) (rice), Wudeli Flour Group Co., Ltd. (Handan, China) (wheat flour), Junan Zhengqiang Peanut Food Co., Ltd. (Junan, China) (peanuts), Feixian Linjia Wuguzaliang She Co., Ltd. (Linyi, China) (white sesame seeds), and Muling Beichun Agricultural Technology Co., Ltd. (Muling, China) (soybeans), respectively. To ensure the purity of the blank matrix, commercially available gluten-free flour was not used. Instead, rice flour was freshly prepared by grinding polished rice using a high-speed grinder (16,000 rpm, 5 min) to obtain a homogeneous powder, which was stored in clean, dry, and sealed containers under dark conditions to prevent moisture absorption and oxidation.

A total of 330 gluten-free flour samples were prepared, including non-contaminated gluten-free flour controls and artificially prepared allergen-containing flour cross-contaminated samples containing wheat, peanut, soybean, and sesame flours at different contamination levels (Table S1). In this study, an independent sample was defined as an independently prepared flour mixture. For each allergen-containing flour category, allergen-containing flour was individually weighed and mixed with gluten-free flour to achieve contamination levels ranging from 1% to 25% (*w*/*w*). This concentration range was selected based on previous NIR-based studies [11,12] and to provide sufficient spectral variation for model developments and evaluations while representing different levels of allergen-containing flour addition relevant to high-level cross-contamination screening. Two independent samples were prepared at each contamination level (1–25% [*w*/*w*], at 1% interval) through separate weighing and mixing procedures, resulting in 50 independently prepared samples per allergen category. In addition, two additional independent samples were prepared at eight selected contamination levels (3%, 6%, 9%, 12%, 15%, 18%, 21%, and 24% [*w*/*w*]), generating 16 additional samples to increase sample diversity and incorporate preparation-related variability. Therefore, each allergen-containing flour category contained 66 independently prepared samples. The gluten-free flour control group consisted of 66 independently prepared samples without allergen addition (0%, *w*/*w*). To ensure balanced representation across allergen-containing flour types and addition levels, a stratified random sampling strategy based on class type and contamination level was applied to divide the dataset into a calibration (training) set (~80%, n = 264) and an independent hold-out validation (test) set (~20%, n = 66). To ensure homogeneity, all samples were thoroughly mixed for 40 min as a standardized preparation procedure. All samples were sealed, labeled, and stored under low-temperature, dry, and dark conditions prior to spectral acquisition.

### 2.2. Spectral Data Acquisition

NIR spectra were collected using a portable Fourier-transform NIR (FT-NIR) spectrometer (Neospectra-Scanner, Si-Ware Systems, Cairo, Egypt). The instrument is equipped with a MEMS-based single-chip Michelson interferometer and an extended indium gallium arsenide (InGaAs) photodetector array. Spectral measurements were acquired over the wavelength range of 1350–2500 nm with a spectral resolution of 16 nm.

Prior to spectral acquisition, the instrument was allowed to warm up and calibrated using a diffuse reflectance reference standard to minimize instrumental drift and environmental variations. Background calibration was repeated every hour throughout the experiment. Samples were analyzed in diffuse reflectance mode. Approximately 2 g of flour was evenly distributed in a 20 mm Petri dish (PerkinElmer, Inc., Shelton, CT, USA) and gently compacted using a solid sample press accessory (Figure 1A) to form a uniform surface without visible cracks or voids. The Petri dish was thoroughly cleaned and wiped with 70% ethanol and Kimwipes between samples to prevent potential cross-contamination. To minimize sampling variability, sample thickness, surface flatness, and packing density were maintained as consistently as possible throughout the study. The acquisition time was set to 10 s to enable rapid analysis while maintaining an acceptable signal-to-noise ratio. For each independent sample, three replicate spectra were acquired and averaged to obtain one representative spectrum. The resulting averaged spectra were subsequently used for dataset splitting, spectral preprocessing, and model development. Between measurements, sample holders were thoroughly cleaned and residual flour particles were removed to prevent cross-contamination. Instrument control and spectral acquisition were performed using the NeoSpectra application on a Bluetooth-connected tablet (Figure 1A). All spectral data were automatically recorded, archived, and compiled into a spectral database for subsequent preprocessing, multivariate data analysis, and model development.

### 2.3. Multivariate Data Analysis

All multivariate data analyses were performed using Pirouette software (Version 5.0, Infometrix Inc., Bothell, WA, USA). Prior to model development, all spectra were mean-centered to reduce systematic offsets and spectral collinearity. Additional preprocessing methods were evaluated to minimize baseline variation, particle scattering effects, and instrumental noise. The optimal preprocessing strategy for each model was selected based on classification and prediction performance. Principal component analysis (PCA) was initially performed as an unsupervised exploratory technique to visualize sample distribution patterns and evaluate spectral variability among uncontaminated and allergen-containing gluten-free flour samples. For PCA, spectra were preprocessed using mean-centering, normalization, smoothing (25-point window), and Savitzky–Golay second-derivative transformation (35-point window). To assess model performance and discrimination capability under the investigated conditions, the dataset was divided into calibration (80%) and independent validation (20%) subsets using stratified random sampling according to contamination type and contamination level for Soft independent modeling of class analogy (SIMCA) and Partial least squares regression (PLSR) analysis.

SIMCA was employed for qualitative classification of allergen-containing flour cross-contamination status. SIMCA is a supervised classification technique based on principal component analysis, in which independent PCA models are developed for each class and unknown samples are classified according to their distances from the corresponding class models. Outlier diagnosis for SIMCA models was performed using the Mahalanobis distance calculated in the score space of each class model. For a sample with score vector t_i_, its Mahalanobis distance from the centroid of the corresponding class model was calculated based on the score covariance matrix. The critical Mahalanobis distance (MDcrit ) was determined from the F-distribution at a significance level of α = 0.05. Samples with Mahalanobis distance values exceeding MDcrit were considered potential outliers [18]. Based on this criterion, two samples from the sesame class were identified as outliers and excluded from subsequent SIMCA model development (Figure S1). Individual SIMCA models were established for rice flour, and wheat-, peanut-, soybean-, and sesame-containing samples to enable identification of different ingredient cross-contamination scenarios. For SIMCA analysis, the same preprocessing procedure used for PCA was applied, including mean-centering, normalization, smoothing (25-point window), and Savitzky–Golay second-derivative transformation (35-point window). Model performance was evaluated using classification accuracy, sensitivity, specificity, and misclassification rate. Class projections and discriminating power plots were used to identify spectral regions contributing most significantly to class separation. Interclass distance (ICD) values were calculated to quantify similarities and differences among classes, with ICD values greater than 3 considered indicative of satisfactory class discrimination.

PLSR was used to establish quantitative models relating NIR spectral responses to allergen-containing flour addition levels (% *w*/*w*). Separate models were developed for wheat, peanut, soybean, and sesame flour contaminations. For PLSR outlier diagnosis, leverage and studentized residuals were examined to identify potentially influential observations. Leverage (hi) was used to assess the distance of each sample from the centroid of the calibration samples in the latent-variable space and was compared with the commonly used criterion hcrit = 2k/n, where k is the number of latent variables and n is the number of calibration samples. Studentized residuals were used to evaluate the magnitude of the response residuals while accounting for the effect of leverage. Pirouette calculated the critical Studentized residual (rcrit) at a 95% probability level. Samples exceeding these diagnostic criteria were further examined for potential influence on the model rather than being automatically excluded. In particular, a high-leverage observation associated with an extreme y value may simply represent a sample located at the edge of the calibration range and should not necessarily be considered an outlier [18]. Therefore, an observation was considered a potential outlier only when both diagnostics indicated abnormal behavior and the observation was determined to have a potentially undue influence on the model. Based on these criteria, no samples were identified as outliers, and therefore no samples were excluded from the PLSR models.

Prior to PLSR modeling, all spectra were first mean-centered. Subsequently, four additional preprocessing strategies were evaluated for PLSR model development, each applied after mean-centering, including Savitzky–Golay second-derivative transformation with a 35-point window, normalization, Savitzky–Golay second-derivative transformation (35-point window) combined with normalization, and Savitzky–Golay second-derivative transformation (35-point window) combined with smoothing (35-point window). For each contamination type, all preprocessing strategies were evaluated using the calibration dataset. Model optimization and cross-validation were performed using two cross-validation approaches, including leave-one-out cross-validation (LOOCV) and leave-10-out cross-validation. Both cross-validation procedures were independently conducted for each PLSR model developed using different preprocessing strategies. The cross-validation results from both approaches were used to compare model performance and statistically evaluate differences among preprocessing strategies using one-way repeated-measures ANOVA followed by Holm-adjusted pairwise comparisons (α = 0.05). The number of latent variables was independently determined within each cross-validation procedure by considering cross-validation prediction error, explained variance, and model complexity to achieve an appropriate balance between predictive performance and model simplicity.

When models showed comparable predictive performance, additional criteria, including the number of latent variables and regression vector characteristics, were considered. Models with comparable accuracy but fewer latent variables and smoother regression vectors were preferred because they provide simpler and more interpretable chemometric models. Based on the above evaluation procedure, soybean-contaminated samples were finally modeled using mean-centering, followed by Savitzky–Golay second-derivative transformation (35-point window) and normalization, whereas wheat-, peanut-, and sesame-contaminated samples were modeled using mean-centering, followed by Savitzky–Golay second-derivative transformation (35-point window) and smoothing (35-point window).

After the selection of the final model, independent hold-out validation subsets (20%) generated using stratified random sampling according to contamination type and contamination level were used for final model evaluation. PLSR model performance was evaluated using the coefficient of determination for calibration (Rcal) and validation (Rval), together with the root mean square error of calibration (RMSEC), root mean square error of cross-validation (RMSECV), and root mean square error of prediction (RMSEP). SIMCA model performance was evaluated using classification accuracy, sensitivity, specificity, and misclassification rate.

## 3. Results and Discussion

### 3.1. Spectral Characterization of Gluten-Free Rice Flour and Allergen-Containing Flour Cross-Contaminated Samples

The raw FT-NIR spectra of gluten-free rice flour and allergen-containing flour cross-contaminated samples are shown in Figure 1B. All samples exhibited broadly similar spectral profiles across the 1350–2500 nm region, regardless of type or contamination level of allergen-containing flour, reflecting the common chemical constituents present in rice flour and the allergenic powders. The spectra contained several broad and overlapping absorption features associated with major food components, including carbohydrates, proteins, lipids, and moisture. The broad absorption features near approximately 1450 and 1940 nm can generally be associated with O-H stretching overtones and combination vibrations, respectively, which are commonly influenced by moisture content and hydroxyl-containing carbohydrates [16]. Absorption features in the region of 1700–1800 nm are typically related to C–H stretching overtones, which may originate from lipids and other organic compounds [19]. The spectral region between 2050 and 2250 nm contains overlapping combination bands involving N–H, C=O, and C–N vibrations, which have been broadly linked to protein-related constituents, including amide groups [20]. Therefore, variations in this region may reflect differences in protein composition among wheat, peanut, soybean, and sesame powders. Additional absorption features observed between 2250 and 2400 nm are generally associated with combinations of C–H stretching and deformation vibrations from multiple macromolecular components, including carbohydrates, fatty acids, and other organic compounds [21,22,23,24].

Prior to multivariate analysis, the raw spectra were preprocessed to improve spectral standardization, reduce baseline variation, and enhance subtle compositional differences among samples. Figure 1C–F illustrates the effects of different preprocessing methods on the FT-NIR spectra of rice flour and contaminated flour samples used for PCA and SIMCA analysis. Mean-centering (Figure 1C) shifted the spectral variables around a common mean, reducing systematic offsets and emphasizing sample-to-sample variation [25]. Normalization (Figure 1D) reduced variations in overall spectral intensity arising from differences in flour packing density, particle size distribution, and diffuse light scattering, resulting in spectra with comparable amplitudes while preserving their characteristic absorption patterns [26]. Subsequent smoothing (Figure 1E) effectively suppressed high-frequency instrumental noise and produced smoother spectral profiles without altering the major absorption features [27]. Finally, application of the Savitzky–Golay second-derivative transformation (Figure 1F) further corrected baseline variations and enhanced spectral resolution by resolving overlapping absorption bands that are common in flour matrices, making subtle compositional differences associated with contaminations more distinguishable [28].

After sequential preprocessing, subtle spectral differences among the different allergen-containing contamination groups became more apparent (Figure 1F). Peanut-contaminated samples exhibited relatively stronger spectral features in the C–H-related regions (approximately 1700–1800 and 2250–2400 nm), which may be attributed to the higher lipid content of peanut flour and the associated C–H stretching overtones and combination bands. In contrast, wheat-contaminated samples showed comparatively weaker responses in these regions, reflecting their lower lipid content [29]. More pronounced variations were observed for soybean- and sesame-contaminated samples within the protein-associated region (approximately 2050–2250 nm), corresponding primarily to the combination vibrations of N–H, C=O, and C–N bonds. These differences are likely related to variations in protein composition and amino acid profiles among the different allergenic flours [30]. In addition, noticeable variations around 1450 and 1940 nm, corresponding to O–H overtone and combination bands, were observed particularly for wheat- and sesame-contaminated samples, which may reflect differences in moisture retention and hydroxyl-containing constituents, including carbohydrates and proteins. Although these spectral differences were relatively subtle, they indicate that composition-related variations introduced by different allergen-containing flour additions are preserved in the FT-NIR spectra, providing the chemical basis for subsequent multivariate classification and quantitative prediction.

### 3.2. Multivariate Pattern Recognition for Classification

PCA was first applied to the preprocessed NIR spectral dataset to explore intrinsic sample distribution patterns and evaluate the natural separability among rice flour and cross-contaminated samples. The 3D PCA score plot (Figure 2A) shows that gluten-free rice flour, wheat-, peanut-, soybean-, and sesame-contaminated samples formed well-defined and compact clusters in the principal component space. The first three principal components (PC1–PC3) accounted for 98.2% of the total spectral variance, effectively capturing the major chemical information embedded in the NIR spectra. A clear clustering pattern was observed, where rice flour and contaminated samples were distinctly separated with minimal overlap, indicating strong inherent spectral discriminability. Among all classes, wheat-contaminated samples showed the closest distribution to rice flour, suggesting relatively higher similarity in their overall macromolecular composition. In contrast, peanut- and sesame-contaminated samples were more distinctly separated from rice flour, which may be attributed to their higher lipid content. Soybean-contaminated samples exhibited an intermediate distribution pattern, reflecting their balanced composition of proteins and lipids compared with the other allergen-containing flour types. Overall, the PCA results demonstrate that the intrinsic spectral variability among different contaminated flour samples is sufficiently large to support subsequent supervised classification modeling.

Based on the PCA framework, SIMCA was employed for classification. SIMCA constructs an individual PCA submodel for each class, enabling class-specific representation of spectral variability and improving discrimination in high-dimensional and highly collinear NIR data. In the SIMCA training model, an optimal number of principal components was selected for each class to capture the majority of spectral variance while avoiding overfitting. Specifically, rice flour and allergenic flour contaminated classes were modeled using an optimal range of latent PCs (6–8 PCs per class), which collectively explained more than 99.6% of the variance within each class. This ensured sufficient information retention while maintaining model compactness and reducing the risk of overfitting, thereby providing effective and well-structured class representations. Overall, all five classes were well separated in the 3D SIMCA projection plot (Figure 2B), with no misclassification observed in the training model, indicating strong inter-class discrimination (Tables S2 and S3). The ICD matrix (Table 1) further confirmed clear separability among classes. All pairwise ICD values between gluten-free rice flour and cross-contaminated samples exceeded the commonly accepted (significantly difference) threshold of 3 (4.7–17.2), demonstrating pronounced spectral dissimilarity and strong class discrimination [31]. Among cross-contaminated classes, peanut and sesame exhibited relatively lower ICD values (1.6), suggesting higher spectral similarity, likely due to comparable lipid- and protein-associated absorption features, although no misclassification was observed. In contrast, the remaining contamination groups showed substantially higher ICD values, indicating stronger inter-class separability. Key discriminative spectral regions identified by the SIMCA training model are shown in the discriminating power plot (Figure 2C). The most significant bands associated with class separation were observed around 1723 nm, corresponding to the first overtone of C–H stretching vibrations, which is particularly prominent in lipid-rich samples such as peanut and sesame. The band near 1657 nm is associated with C–H stretching vibrations of protein backbones and side chains, reflecting compositional differences among wheat (gluten proteins), soybean (globulins), and sesame proteins [32,33].

The predictive performance of the SIMCA model built from NIR spectral data was evaluated using an independent hold-out validation set (n = 65, 13 samples in each class) comprising 20% of the total samples (Tables S2 and S3). As shown in Figure 2D, all the validation samples were accurately predicted into their actual class, demonstrating 100% accuracy in the prediction performance. Sensitivity determined the ability of the model to correctly identify samples belonging to each specific class of gluten-free rice flour and cross-contaminated samples. Specificity reflected the ability of the model to correctly reject samples that did not belong to the target class [34]. The SIMCA independent hold-out validation results indicated 100% sensitivity (true positive n = 65, false negative n = 0) and 100% specificity (false positive n = 0, true negative = 65) across all classes (Table 2), demonstrating excellent classification performance and feasibility of the proposed sensing strategy for independent sample prediction under controlled conditions. Previous studies have demonstrated the potential of NIR spectroscopy combined with chemometrics for allergen-related analysis and food-material discrimination under different experimental conditions. For example, Rady et al. used a compact NIR sensor (1550–1950 nm) to classify different allergen-containing powdered foods, including gluten-containing flours, gluten-free flours, nuts, and animal-based powders, with classification accuracies reaching 100% under optimized conditions [35]. Rady and Watson investigated peanut contamination in garlic powder using low-cost NIR sensors covering 1550–1950 nm and 2000–2450 nm, with contamination levels ranging from 0.01% to 20%, and reported a classification accuracy of 85.1% using samples from a different origin [36]. These studies employed different experimental designs, ranging from discrimination among different allergen-containing ingredients to detection of single-allergen contamination, with reported classification accuracies generally exceeding 85%. In the present study, the MEMS-based NeoSpectra-Scanner covers a relatively broad spectral range of 1350–2500 nm, providing access to information-rich spectral features across the near-infrared region. The proposed handheld NIR-based SIMCA model effectively discriminated among four allergen-specific single-contaminant scenarios and the gluten-free control across the investigated concentration range (0% control vs. 1–25% contamination, w/w), demonstrating reliable performance within the investigated laboratory dataset.

### 3.3. PLSR-Based Multivariate Modeling for Allergen-Containing Flour Contamination Level Prediction

Once the classification algorithm determines whether the gluten-free flour is contaminated and identifies the type of allergen-containing flour involved, further prediction of the allergen-containing flour cross-contamination level is important for assessing the extent of cross-contact. Therefore, PLSR models were developed using handheld FT-NIR spectral data and the corresponding known allergen-containing flour cross-contamination levels (% *w*/*w*).

Prior to PLSR modeling, all spectra were first mean-centered. Subsequently, four additional preprocessing strategies were systematically evaluated using the calibration dataset, each applied after mean-centering, including Savitzky–Golay second-derivative transformation (35-point window), normalization, Savitzky–Golay second-derivative transformation combined with normalization, and Savitzky–Golay second-derivative transformation combined with smoothing (35-point window). The preprocessing strategies were compared using cross-validation performance obtained from LOOCV (Figures S2–S5) and leave-10-out cross-validation (Figures S6–S9). Since statistical comparisons showed no consistent significant differences among preprocessing approaches (Table S4), the final preprocessing strategy was not selected solely based on numerical differences in prediction errors. Instead, when comparable predictive performance was observed, model complexity and spectral interpretability were considered, including the number of latent variables and regression vector characteristics.

For soybean-contaminated samples (Figure S2), mean-centering followed by Savitzky–Golay second-derivative transformation and normalization provided a favorable balance between predictive performance and model interpretability. This preprocessing strategy achieved relatively low RMSECV with only four latent variables under LOOCV evaluation compared with alternative approaches. Moreover, the corresponding regression vector showed relatively clear spectral contribution patterns without excessive complexity, supporting the selection of this preprocessing strategy for soybean-contaminated samples.

For wheat-, peanut-, and sesame-contaminated samples (Figures S3–S5), the normalization-based approaches did not provide clear predictive advantages and generally required more latent variables, resulting in increased model complexity. Although Savitzky–Golay second-derivative transformation alone showed slightly lower prediction errors in some cases, the corresponding regression vectors exhibited more complex spectral patterns. In contrast, Savitzky–Golay second-derivative transformation followed by smoothing maintained comparable predictive performance while providing smoother and more interpretable regression vectors. Considering model simplicity, regression vector stability, and the broad spectral features typically observed in NIR spectroscopy, mean-centering followed by Savitzky–Golay second-derivative transformation and smoothing was selected as the final preprocessing strategy. The performance statistics of the final PLSR models developed using the selected preprocessing strategies and the calibration dataset (80% of the total samples), including LOOCV-based cross-validation results, and independent hold-out validation (20%) are summarized in Table 3.

Soybean is a common allergenic ingredient and is frequently involved in cross-contamination during food processing. The soybean PLSR model was developed using four latent variables, resulting in a compact and stable model structure that explained 97.88% of the total spectral variance (Table 3 and Figure 3A). Excellent calibration performance was achieved (Rcal = 0.99), indicating a strong linear relationship between spectral data and concentration levels within the investigated laboratory dataset. The RMSEC (0.95) and RMSECV (1.01) values remained low and comparable, confirming good model fitting accuracy and internal consistency. Independent hold-out validation further demonstrated effective predictive capability (Rval = 0.99, RMSEP = 1.22), with consistent agreement between calibration and prediction results. Regression vectors are commonly used to evaluate the relative contribution of each variable to the response of interest, thereby facilitating the interpretation of the spectral regions associated with allergen-containing flour cross-contamination level [37]. Key spectral features were at 1723 and 2331 nm (Figure 3B) attributed to N–H/C–H combination vibrations and protein backbone overtones, mainly originating from soybean globulin structures. These bands reflect both protein and minor lipid contributions, consistent with the high-protein composition of soybean flour [38].

The peanut PLSR model employed four latent variables, which explained 99.39% of the total spectral variance (Table 3 and Figure 4A). Excellent calibration performance was obtained (Rcal = 0.99), indicating a strong linear relationship between spectral responses and contamination levels within the investigated laboratory dataset. The independent hold-out prediction performance (Rval = 0.97, RMSEP = 1.62) was slightly lower than that of the calibration set, suggesting increased variability in independent samples. This discrepancy may be attributed to heterogeneous peanut particle size distribution, non-uniform mixing within the flour matrix, and pronounced lipid-related scattering effects inherent to peanut flour. Despite this, the overall prediction trend remained stable without systematic deviation, confirming the reliable performance of the model for screening applications within the investigated laboratory dataset. The key spectral regions contributing to the successful development of the PLSR model (Figure 4B) were mainly associated with lipid- and protein-related vibrational modes. The band at 1707 nm is attributed to combination vibrations of C–H stretching modes in lipids, reflecting the high lipid content of peanut flour. The feature at 2198 nm is assigned to combination vibrations of N–H stretching and bending modes in proteins, corresponding to amide-related absorption bands. Overall, the PLSR model does not directly quantify total protein content. Instead, it captures subtle variations in protein conformation, side-chain structure, and hydration state across samples, which are indirectly associated with changes in peanut contamination levels [39].

Wheat protein residues represent the primary allergenic component in gluten-free foods, and their accurate quantification is critical for product integrity and safety. The PLSR model for wheat contamination was constructed using seven latent variables, which explained 99.61% of the total variance, indicating sufficient information extraction while minimizing the risk of overfitting (Table 3). The relatively large number of latent variables reflects the high spectral similarity between wheat flour and gluten-free flour matrices, requiring more factors to resolve subtle compositional differences. This is also consistent with the SIMCA results, which showed that gluten-free flour had the smallest distance to the wheat-contaminated class among all evaluated classes. The calibration performance was strong, with Rcal = 0.98, RMSEC = 1.54, and RMSECV = 1.96. The close agreement between calibration and cross-validation errors indicates good model stability and no evident overfitting. Independent hold-out validation further confirmed effective predictive performance within the investigated experimental conditions, yielding a correlation coefficient of 0.96 and an RMSEP of 2.22. As shown in the prediction plot (Figure 5A), both calibration and test samples were evenly distributed around the regression line, with no obvious outliers or systematic bias, demonstrating strong discrimination ability within the investigated laboratory dataset. The key wavelength regions identified by the PLSR model (Figure 5B) were centered at 2400 and 2045 nm. These bands are mainly associated with N–H and C–N combination vibrations of peptide bonds, as well as C–H stretching and deformation combination modes, which are characteristic of gluten protein structures [11,40,41,42]. These results indicate that the model captures chemically meaningful information directly related to gluten composition, supporting the quantification of wheat contamination in gluten-free flour systems.

The sesame PLSR model exhibited excellent overall performance (Table 3 and Figure 6A) among the four contamination types, requiring only four latent variables to explain 96.29% of the total variance while achieving high predictive accuracy within the investigated laboratory dataset. The calibration and cross-validation coefficients were 0.99 and 0.98, respectively, indicating strong internal consistency and no evidence of overfitting. The low RMSEC (1.05) and RMSECV (1.15) further supported the reliable model performance, while independent hold-out validation confirmed strong predictive performance (R = 0.98, RMSEP = 1.37), demonstrating effective discrimination ability. The key spectral features (2324, 2205, 2098, and 1703 nm; Figure 6B) were attributed to amide-related vibrations and lipid-associated C–H combination bands, consistent with the patterns observed in peanut samples and in agreement with the SIMCA projection results. These spectral signatures reflect the coupled protein–lipid structural characteristics of sesame, enabling accurate quantification with a minimal number of latent variables [34,43].

A comparative analysis of the four PLSR models (wheat, soybean, peanut, and sesame) demonstrates consistently strong calibration and predictive performance under the investigated datasets, confirming the feasibility of handheld NIR spectroscopy for rapid estimation of allergen-containing flour cross-contamination levels in gluten-free flour systems. All models achieved high calibration performance (Rcal > 0.98), with independent hold-out prediction accuracy remaining above 0.96, indicating stable and reliable quantitative relationships between spectral features and allergen-containing flour cross-contamination levels (% *w*/*w*). Differences in model performance among contamination types are primarily associated with compositional variability. In particular, sesame, peanut, and soybean models are more strongly influenced by lipid content compared with wheat, which contributes to increased spectral complexity and matrix effects. Compared with previously reported studies for allergenic ingredient cross-contamination screening, Wu et al. investigated the detection and quantification of artificially introduced wheat, peanut, and sesame in quinoa flour (0–98%) using NIR spectroscopy combined with PLSR [11]. The benchtop NIR model achieved an Rval^2^ of approximately 0.99 and an RMSEP of 3.25, while the filter-based NIR model achieved an Rval^2^ of approximately 0.96 and an RMSEP of 6.32. Under the investigated contamination range of 1–25%, which represents a narrower range compared with the previously reported study (0–98%), the proposed handheld NIR-based model demonstrated reliable predictive capability for multi-class discrimination and contamination-level estimation among four single-ingredient contamination scenarios (wheat, soybean, peanut, and sesame) in gluten-free flour under the investigated laboratory conditions. Although the internal hold-out validation set demonstrated high classification performance within the investigated laboratory dataset, the relatively limited compositional variability of the raw materials represents an important limitation of the present feasibility study. The use of materials from a limited number of suppliers and a single production batch may not adequately capture the compositional heterogeneity encountered across commercial products and production environments. Such variability, including differences in physicochemical properties and matrix composition, may introduce additional spectral variation and potentially affect the generalizability and transferability of the developed models. Therefore, further validation using samples collected from diverse sources and production environments is necessary before broader practical implementation. Future studies will focus on expanding the dataset to include samples from multiple production batches and suppliers, with broader physicochemical properties (e.g., moisture content and particle-size distribution), processing conditions, and naturally occurring contamination scenarios. Systematic robustness evaluations under varying instruments and environmental conditions, together with external validation using independent industrial samples, will also be conducted to further assess the generalizability, robustness, and practical applicability of the developed models.

This current handheld NIR approach would be suitable as a rapid, non-destructive screening tool for relatively high-level allergen cross-contamination (1-25%, w/w), such as preliminary raw-material screening, in-process monitoring, or rapid screening following potential cross-contact events in gluten-free production. Samples requiring regulatory-level confirmation or low-level allergen-specific quantification would still require validated allergen-specific analytical methods. Future studies will extend the experimental range toward substantially lower flour addition levels and relevant regulatory thresholds, while incorporating allergen-specific reference measurements (e.g., ELISA or LC-MS/MS) to establish the relationship between flour addition level and actual allergenic protein/component concentrations and to determine the corresponding analytical performance, including the limit of detection and limit of quantification.

## 4. Conclusions

Cross-contamination involving allergen-containing ingredients poses a critical challenge to the food industry and product safety. In this study, a rapid screening and quantification strategy was developed for discriminating different allergen contamination categories and predicting contamination levels in gluten-free flour using handheld near-infrared spectroscopy integrated with multivariate data analysis. Under the investigated laboratory conditions, the proposed approach achieved high classification performance (accuracy = 100%) and accurate quantitative prediction (Rval > 0.96) within the evaluated dataset. The observed performance was associated with spectral differences arising from compositional variations between gluten-free flour and allergen-containing ingredients. The proposed approach enables rapid (~10 s), non-destructive, and portable screening of allergen-containing flour cross-contamination, with promising classification and estimation performance within the investigated laboratory dataset. The approach may provide a practical tool for preliminary screening and in-process monitoring of relatively high-level cross-contamination in gluten-free flour (1% to 25%, w/w). However, the current findings are based on a laboratory dataset with relatively limited variability in raw material sources and compositions, and the generalizability of the developed models requires further validation. In addition, regulatory-level detection and allergen-specific quantification will require evaluation at lower flour addition levels using more diverse samples from multiple suppliers and production batches and broader production environments, together with established allergen-specific reference methods such as ELISA or LC-MS/MS.

## Figures and Tables

**Figure 1:**
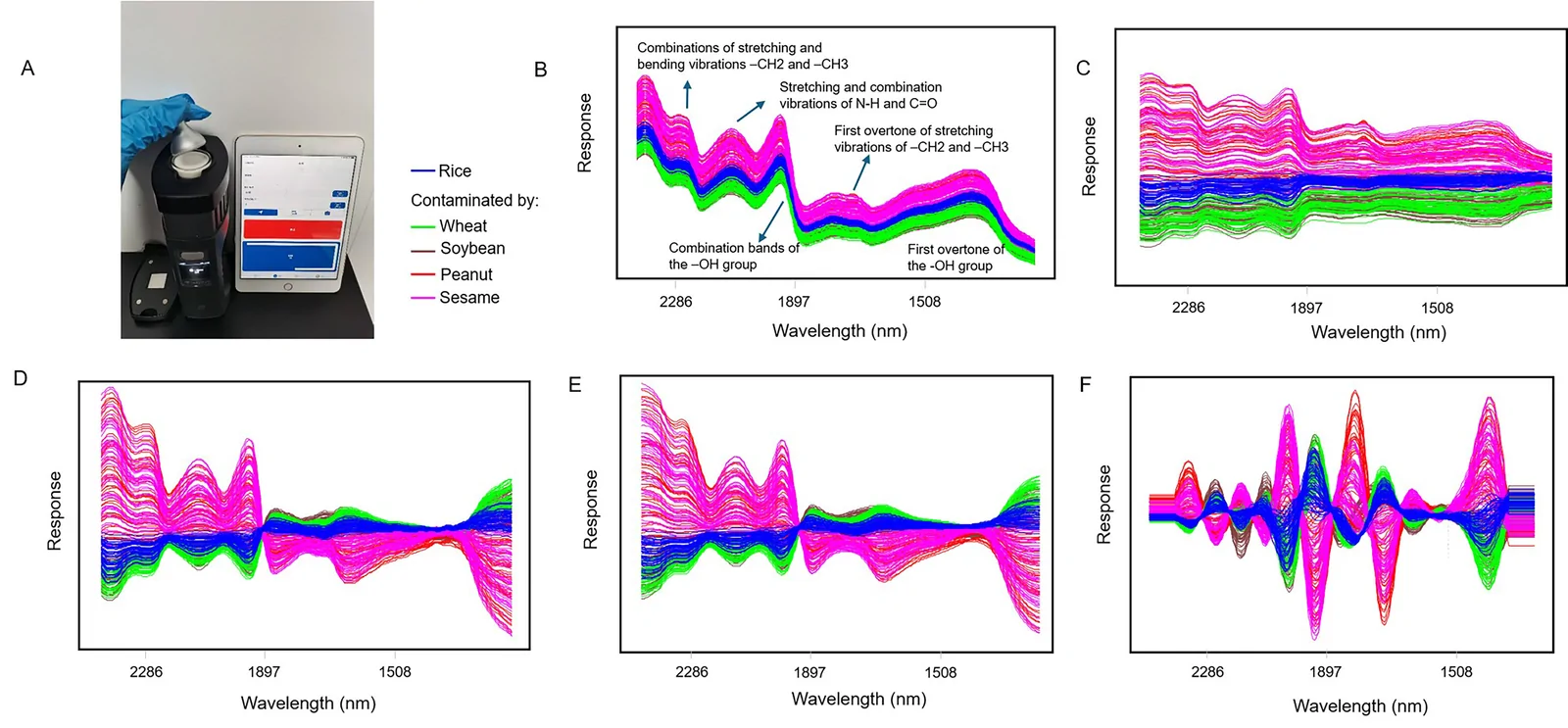
(**A**) Schematic illustration of the handheld FT-NIR spectral acquisition system. (**B**) Raw FT-NIR spectra of gluten-free rice flour and cross-contaminated flour samples. (**C**–**F**) Sequential spectral preprocessing of the FT-NIR spectra: (**C**) mean-centering; (**D**) mean-centering followed by normalization; (**E**) mean-centering, normalization, and smoothing (25-point window); and (**F**) mean-centering, normalization, smoothing (25-point window), and Savitzky–Golay second-derivative transformation (35-point window).

**Figure 2:**
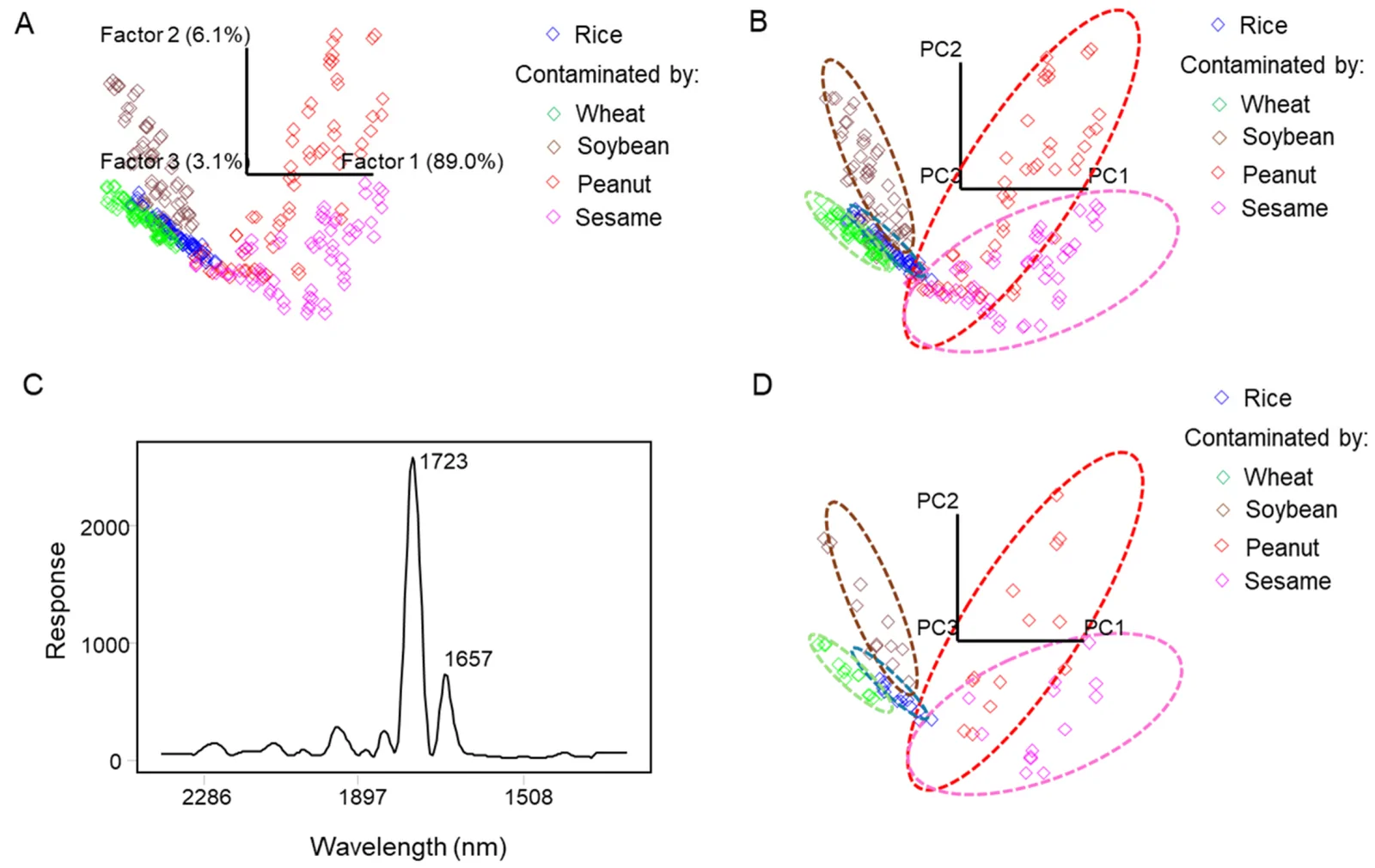
Classification of gluten-free rice flour and cross-contaminated samples. (**A**) PCA score plot; (**B**) 3D SIMCA training model projection of sample distributions; (**C**) SIMCA discriminating power plot; (**D**) Independent hold-out validation results of the SIMCA model.

**Figure 3:**
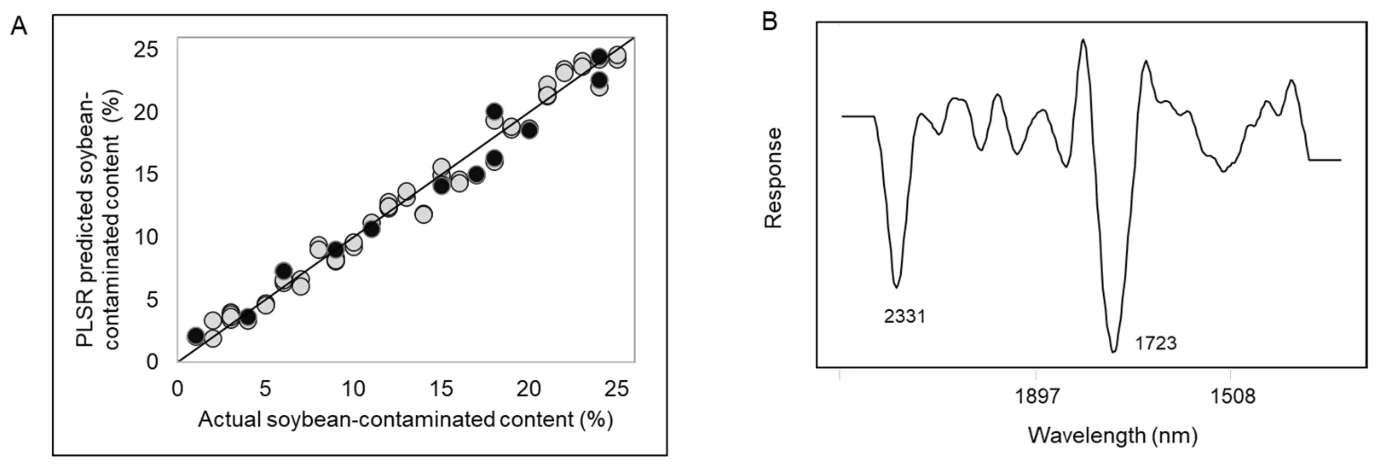
(**A**) PLSR calibration and independent hold-out validation model for soybean-contaminated gluten-free flour (gray circles: calibration samples; black circles: independent hold-out validation samples) and (**B**) its corresponding regression vector plot for spectral interpretation.

**Figure 4:**
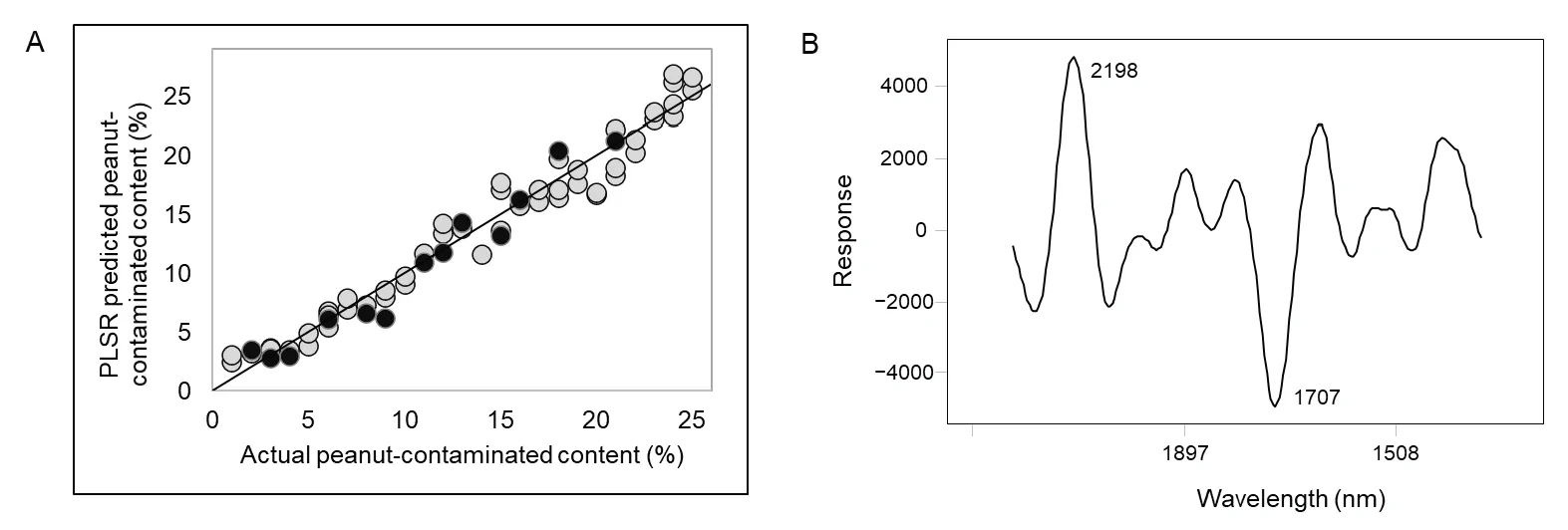
(**A**) PLSR calibration and independent hold-out validation model for peanut-contaminated gluten-free flour (gray circles: calibration samples; black circles: independent hold-out validation samples) and (**B**) its corresponding regression vector plot for spectral interpretation.

**Figure 5:**
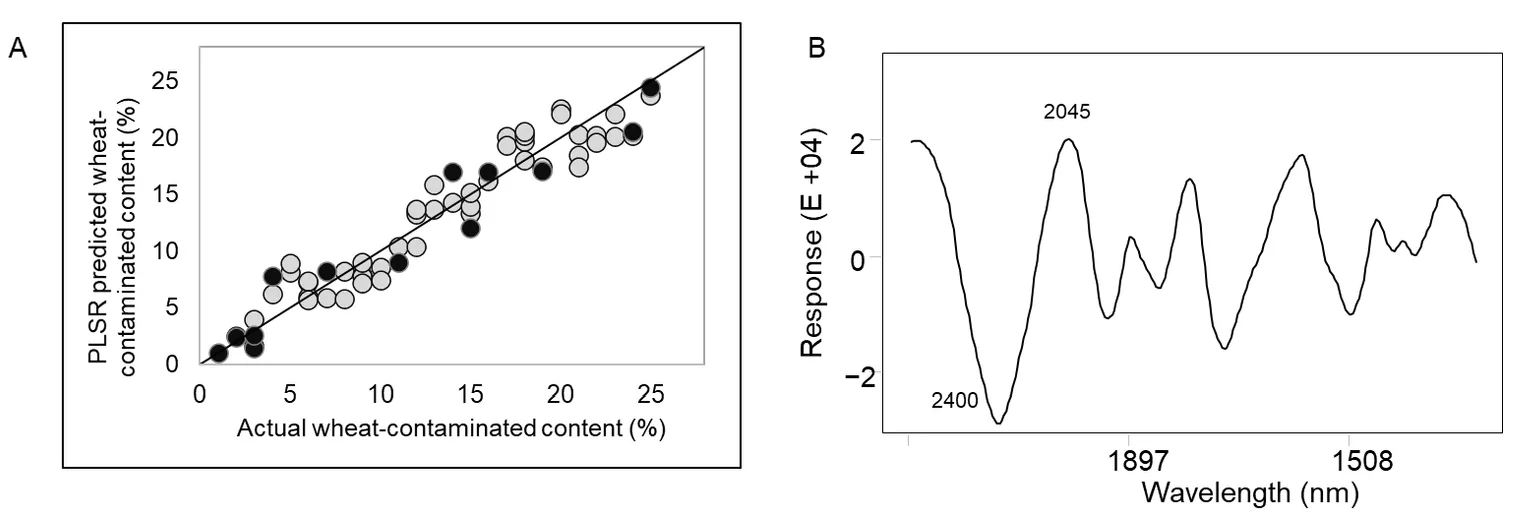
(**A**) PLSR calibration and independent hold-out validation model for wheat-contaminated gluten-free flour (gray circles: calibration samples; black circles: independent hold-out validation samples) and (**B**) its corresponding regression vector plot for spectral interpretation.

**Figure 6:**
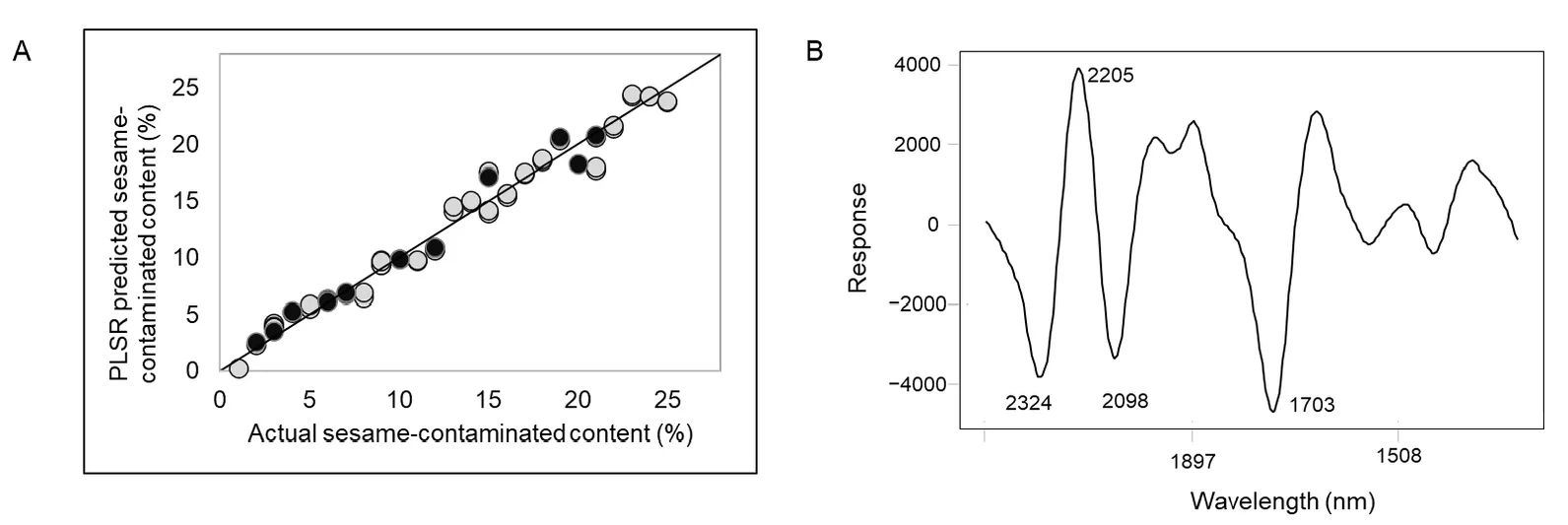
(**A**) PLSR calibration and independent hold-out validation model for sesame-contaminated gluten-free flour (gray circles: calibration samples; black circles: independent hold-out validation samples) and (**B**) its corresponding regression vector plot for spectral interpretation.

**Table 1:** Interclass distance between gluten-free rice flour and four cross-contaminated samples based on SIMCA modeling of NIR spectra (1350–2500 nm).

Groups	Soybean Contamination	Peanut Contamination	Wheat Contamination	Sesame Contamination	Gluten-Free Rice Flour
Soybean-contaminated	0.0				
Peanut-contaminated	5.2	0.0			
Wheat-contaminated	8.0	18.6	0.0		
Sesame-contaminated	3.9	1.6	12.1	0.0	
Gluten-free rice flour	8.4	17.2	4.7	11.6	0.0

**Table 2:** Statistical performance of the SIMCA classification model based on handheld NIR spectral data.

Model	Accuracy (%)	Sensitivity (%)	Specificity (%)
**NIR**	100	100	100

**Table 3:** Statistical performance of the PLSR models for predicting allergen-containing flour cross-contamination levels based on handheld NIR spectral data.

Contaminations	Factor	Range (%)	Training Model (LOOCV)				Independent Hold-Out Validation		
			N ^a^	Rcal ^b^	RMSEC ^c^	RMSECV ^d^	N ^e^	Rval ^f^	RMSEP ^g^
Soybean	4	1–25	53	0.99	0.95	1.01	13	0.99	1.22
Peanut	5	1–25	53	0.99	0.97	1.23	13	0.97	1.62
Wheat	7	1–25	53	0.98	1.54	1.96	13	0.96	2.22
Sesame	4	1–25	53	0.99	1.05	1.15	13	0.98	1.37

^a^ Sample number in the training models. ^b^ Coefficient of determination for calibration. ^c^ Root mean square error of calibration. ^d^ Root mean square error of cross-validation. ^e^ Sample number in the independent hold-out validation models. ^f^ Coefficient of determination for validation set. ^g^ Root mean square error of prediction.

## Data Availability

The data presented in this study are available on request from the corresponding author.

## Supplementary Materials

The following supporting information can be downloaded at: https://www.mdpi.com/article/10.3390/foods15193434/s1, Figure S1: Mahalanobis distance-based outlier diagnosis for the SIMCA model of sesame-containing samples; Figure S2: Selection of the optimal number of latent variables by leave-one-out cross-validation (LOOCV) and corresponding regression vectors for the soybean PLSR models using different spectral preprocessing strategies, each applied after mean-centering; Figure S3: Selection of the optimal number of latent variables by leave-one-out cross-validation (LOOCV) and corresponding regression vectors for the peanut PLSR models using different spectral preprocessing strategies,each applied after mean-centering; Figure S4: Selection of the optimal number of latent variables by leave-one-out cross-validation (LOOCV) and corresponding regression vectors for the wheat PLSR models using different spectral preprocessing strategies,each applied after mean-centering; Figure S5: Selection of the optimal number of latent variables by leave-one-out cross-validation (LOOCV) and corresponding regression vectors for the sesame PLSR models using different spectral preprocessing strategies,each applied after mean-centering; Figure S6: Selection of the optimal number of latent variables by leave-10-out cross-validation and corresponding regression vectors for the soybean PLSR models using different spectral preprocessing strategies,each applied after mean-centering; Figure S7: Selection of the optimal number of latent variables by leave-10-out cross-validation and corresponding regression vectors for the peanut PLSR models using different spectral preprocessing strategies,each applied after mean-centering; Figure S8: Selection of the optimal number of latent variables by leave-10-out cross-validation and corresponding regression vectors for the wheat PLSR models using different spectral preprocessing strategies,each applied after mean-centering; Figure S9: Selection of the optimal number of latent variables by leave-10-out cross-validation and corresponding regression vectors for the sesame PLSR models using different spectral preprocessing strategies,each applied after mean-centering; Table S1: Summary of sample preparation, contamination levels, and dataset allocation; Table S2: Confusion matrices of the SIMCA model for calibration/training and validation sets (A) Calibration/training set (n = 53 samples per class); Table S3: Class-specific performance metrics of the SIMCA model for calibration/training and independent validation sets; Table S4: Statistical ANOVA analysis of PLSR models with different spectral preprocessing strategies based on cross-validation after mean-centering.
