# Supplementary material for: Handheld Near-Infrared Spectroscopy-Based Intelligent Screening of Allergen-Containing Flour Cross-Contamination in Gluten-Free Flour
Source: Foods. 2026 Sep 25;15(19):3434. doi: 10.3390/foods15193434 (PMC13664017; doi:10.3390/foods15193434)

Table S1. Summary of sample preparation, contamination levels, and dataset allocation.

| Sample class | Contamination level (% w/w) | Sample preparation strategy | Number of independent samples | Calibration set | Validation set |
|---|---|---|---|---|---|
| Gluten-free flour control | 0% | Gluten-free flour without allergen addition | 66 | 53 | 13 |
| Wheat contamination | 1-25% | Two independent mixtures prepared at each contamination level (50 samples); additional two independent mixtures prepared at selected levels (16 samples) | 66 | 53 | 13 |
| Peanut contamination | 1-25% | Two independent mixtures prepared at each contamination level (50 samples); additional two independent mixtures prepared at selected levels (16 samples) | 66 | 53 | 13 |
| Soybean contamination | 1-25% | Two independent mixtures prepared at each contamination level (50 samples); additional two independent mixtures prepared at selected levels (16 samples) | 66 | 53 | 13 |
| Sesame contamination | 1-25% | Two independent mixtures prepared at each contamination level (50 samples); additional two independent mixtures prepared at selected levels (16 samples) | 66 | 53 | 13 |
| Total | 0-25% | — | 330 | 265 | 65 |

Each contamination level ranged from 1% to 25% (w/w) at 1% intervals.

Selected contamination levels included 3%, 6%, 9%, 12%, 15%, 18%, 21%, and 24% (w/w).

Table S2. Confusion matrices of the SIMCA model for calibration/training and validation sets

(A) Calibration/training set (n = 53 samples per class)

| Actual contamination class / Predicted contamination class | Control | Wheat | Peanut | Soybean | Sesame |
|---|---|---|---|---|---|
| Control | 53 | 0 | 0 | 0 | 0 |
| Wheat | 0 | 53 | 0 | 0 | 0 |
| Peanut | 0 | 0 | 53 | 0 | 0 |
| Soybean | 0 | 0 | 0 | 53 | 0 |
| Sesame | 0 | 0 | 0 | 0 | 51 |

(B) Validation set (n = 13 samples per class)

| Actual contamination class / Predicted contamination class | Control | Wheat | Peanut | Soybean | Sesame |
|---|---|---|---|---|---|
| Control | 13 | 0 | 0 | 0 | 0 |
| Wheat | 0 | 13 | 0 | 0 | 0 |
| Peanut | 0 | 0 | 13 | 0 | 0 |
| Soybean | 0 | 0 | 0 | 13 | 0 |
| Sesame | 0 | 0 | 0 | 0 | 13 |

Table S3. Class-specific performance metrics of the SIMCA model for calibration/training and independent validation sets.

| Dataset | Class | Sensitivity (%) | Specificity (%) | Precision (%) | Balanced accuracy (%) |
|---|---|---|---|---|---|
| Calibration/training | Control | 100 | 100 | 100 | 100 |
| Calibration/training | Wheat | 100 | 100 | 100 | 100 |
| Calibration/training | Peanut | 100 | 100 | 100 | 100 |
| Calibration/training | Soybean | 100 | 100 | 100 | 100 |
| Calibration/training | Sesame | 100 | 100 | 100 | 100 |
| Validation | Control | 100 | 100 | 100 | 100 |
| Validation | Wheat | 100 | 100 | 100 | 100 |
| Validation | Peanut | 100 | 100 | 100 | 100 |
| Validation | Soybean | 100 | 100 | 100 | 100 |
| Validation | Sesame | 100 | 100 | 100 | 100 |

Table S4. Statistical ANOVA analysis of PLSR models with different spectral preprocessing strategies based on cross-validation after mean-centering.

| Cross-validation method | Allergen | Models compared | ANOVA F-value | p-value (α = 0.05) | Holm-adjusted post-hoc comparison |
|---|---|---|---|---|---|
| LOOCV | Wheat | 2nd derivative; Normalization; 2nd derivative + normalization; 2nd derivative + smoothing | 0.556 | 0.645 | None |
| LOOCV | Peanut | 2nd derivative; Normalization; 2nd derivative + normalization; 2nd derivative + smoothing | 3.635 | 0.014 | None |
| LOOCV | Soybean | 2nd derivative; Normalization; 2nd derivative + normalization; 2nd derivative + smoothing | 0.790 | 0.501 | None |
| LOOCV | Sesame | 2nd derivative; Normalization; 2nd derivative + normalization; 2nd derivative + smoothing | 0.517 | 0.671 | None |
| Leave-10-out CV | Wheat | 2nd derivative; Normalization; 2nd derivative + normalization; 2nd derivative + smoothing | 0.577 | 0.631 | None |
| Leave-10-out CV | Peanut | 2nd derivative; Normalization; 2nd derivative + normalization; 2nd derivative + smoothing | 0.198 | 0.898 | None |
| Leave-10-out CV | Soybean | 2nd derivative; Normalization; 2nd derivative + normalization; 2nd derivative + smoothing | 1.321 | 0.270 | None |
| Leave-10-out CV | Sesame | 2nd derivative; Normalization; 2nd derivative + normalization; 2nd derivative + smoothing | 0.577 | 0.631 | None |

Note: The four PLSR models compared in each analysis used the following spectral preprocessing strategies, each applied after mean-centering: second derivative, normalization, second derivative combined with normalization, and second derivative combined with smoothing. Statistical comparisons were performed using one-way repeated-measures ANOVA based on sample-level cross-validated absolute prediction errors (|predicted − measured|). Statistical significance was evaluated at α = 0.05. Pairwise comparisons were performed using Holm-adjusted correction for multiple comparisons. “None” indicates that no pairwise comparison remained statistically significant after correction.

Figure S1. Mahalanobis distance-based outlier diagnosis for the SIMCA model of sesame-containing samples. The vertical green line represents the critical Mahalanobis distance (MDcrit) at the 95% confidence level. Samples located beyond this boundary were identified as potential outliers.

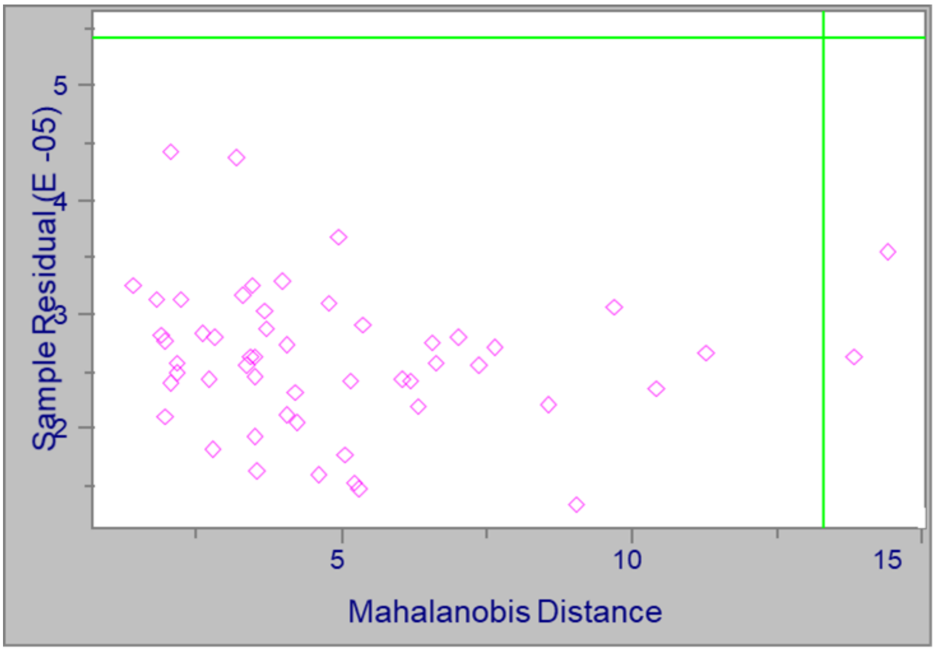

Figure S2. Selection of the optimal number of latent variables by leave-one-out cross-validation (LOOCV) and corresponding regression vectors for the soybean PLSR models using different spectral preprocessing strategies, each applied after mean-centering. For each preprocessing strategy, the three panels from left to right present the RMSECV plot against the number of latent variables (factors), the corresponding explained variance and statistical performance, and the regression vector, respectively. The optimal number of latent variables is highlighted in gray. Panels A–C correspond to second derivative preprocessing (35-point window), panels D–F to normalization, panels G–I to second derivative (35-point window) combined with normalization, and panels J–L to second derivative (35-point window) combined with smoothing (35-point window). Note: r Cal=1.00 represents a rounded value; the actual value was slightly below 1.00.

A

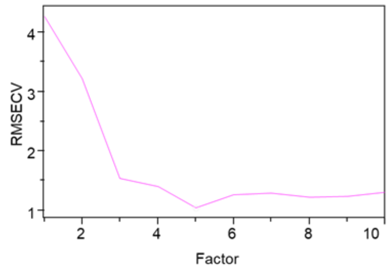


B

| | Variance Percent | Cumulative | RMSEC | RMSECV | r Cal |
|---|---|---|---|---|---|
| Factor1 | 75.24 | 75.24 | 4.19 | 4.27 | 0.82 |
| Factor2 | 10.79 | 86.03 | 3.02 | 3.21 | 0.91 |
| Factor3 | 4.92 | 90.95 | 1.35 | 1.54 | 0.98 |
| Factor4 | 3.63 | 94.58 | 1.26 | 1.39 | 0.99 |
| Factor5 | 4.92 | 99.50 | 0.95 | 1.03 | 0.99 |
| Factor6 | 0.03 | 99.54 | 0.87 | 1.26 | 0.99 |
| Factor7 | 0.11 | 99.64 | 0.81 | 1.28 | 0.99 |
| Factor8 | 0.06 | 99.71 | 0.77 | 1.22 | 1.00 |
| Factor9 | 0.05 | 99.75 | 0.69 | 1.24 | 1.00 |
| Factor10 | 0.03 | 99.78 | 0.61 | 1.30 | 1.00 |

C

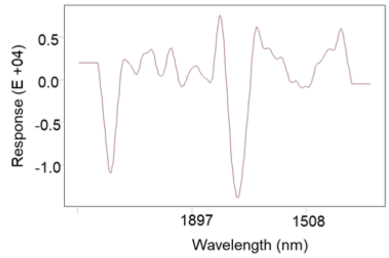


D

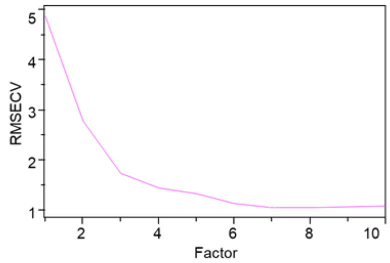


E

| | Variance Percent | Cumulative | RMSEC | RMSECV | r Cal |
|---|---|---|---|---|---|
| Factor1 | 97.24 | 97.24 | 4.83 | 4.88 | 0.75 |
| Factor2 | 0.95 | 98.20 | 2.50 | 2.78 | 0.94 |
| Factor3 | 0.93 | 99.13 | 1.57 | 1.73 | 0.98 |
| Factor4 | 0.23 | 99.36 | 1.31 | 1.44 | 0.98 |
| Factor5 | 0.41 | 99.77 | 1.21 | 1.33 | 0.99 |
| Factor6 | 0.09 | 99.86 | 0.96 | 1.13 | 0.99 |
| Factor7 | 0.01 | 99.87 | 0.88 | 1.05 | 0.99 |
| Factor8 | 0.09 | 99.97 | 0.76 | 1.04 | 1.00 |
| Factor9 | 0.01 | 99.97 | 0.66 | 1.07 | 1.00 |
| Factor10 | 0.00 | 99.98 | 0.53 | 1.08 | 1.00 |

F

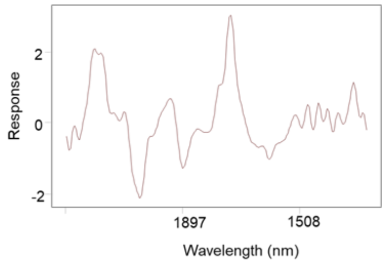


G

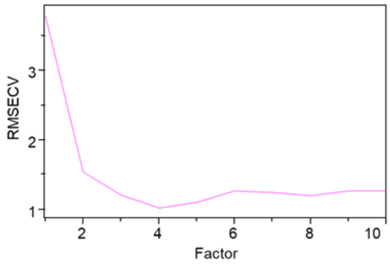


H

| | Variance Percent | Cumulative | RMSEC | RMSECV | r Cal |
|---|---|---|---|---|---|
| Factor1 | 46.39 | 46.39 | 3.61 | 3.78 | 0.87 |
| Factor2 | 20.57 | 66.96 | 1.41 | 1.55 | 0.98 |
| Factor3 | 5.05 | 72.01 | 1.18 | 1.21 | 0.99 |
| Factor4 | 25.88 | 97.88 | 0.95 | 1.01 | 0.99 |
| Factor5 | 0.29 | 98.17 | 0.90 | 1.11 | 0.99 |
| Factor6 | 0.50 | 98.67 | 0.81 | 1.28 | 0.99 |
| Factor7 | 0.26 | 98.93 | 0.77 | 1.24 | 1.00 |
| Factor8 | 0.11 | 99.04 | 0.69 | 1.20 | 1.00 |
| Factor9 | 0.14 | 99.18 | 0.64 | 1.27 | 1.00 |
| Factor10 | 0.12 | 99.30 | 0.58 | 1.27 | 1.00 |

I

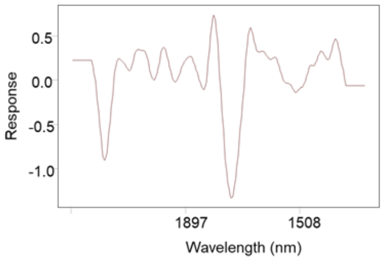


J

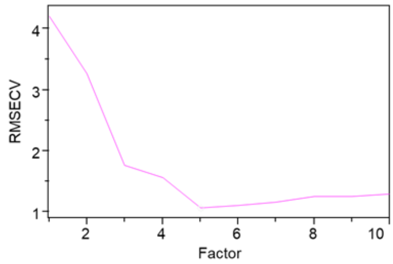


K

| | Variance Percent | Cumulative | RMSEC | RMSECV | r Cal |
|---|---|---|---|---|---|
| Factor1 | 70.46 | 70.46 | 4.13 | 4.21 | 0.82 |
| Factor2 | 14.00 | 84.46 | 3.09 | 3.26 | 0.91 |
| Factor3 | 6.01 | 90.47 | 1.57 | 1.76 | 0.98 |
| Factor4 | 6.62 | 97.08 | 1.39 | 1.55 | 0.98 |
| Factor5 | 2.50 | 99.59 | 0.97 | 1.05 | 0.99 |
| Factor6 | 0.09 | 99.67 | 0.94 | 1.10 | 0.99 |
| Factor7 | 0.09 | 99.76 | 0.92 | 1.15 | 0.99 |
| Factor8 | 0.08 | 99.84 | 0.89 | 1.24 | 0.99 |
| Factor9 | 0.02 | 99.87 | 0.86 | 1.25 | 0.99 |
| Factor10 | 0.02 | 99.89 | 0.82 | 1.29 | 0.99 |

L

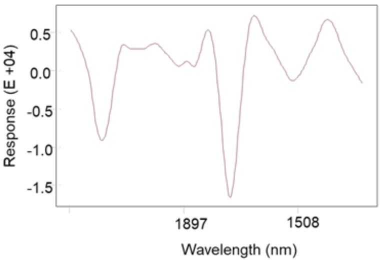

Figure S3. Selection of the optimal number of latent variables by leave-one-out cross-validation (LOOCV) and corresponding regression vectors for the peanut PLSR models using different spectral preprocessing strategies, each applied after mean-centering. For each preprocessing strategy, the three panels from left to right present the RMSECV plot against the number of latent variables (factors), the corresponding explained variance and statistical performance, and the regression vector, respectively. The optimal number of latent variables is highlighted in gray. Panels A–C correspond to second derivative preprocessing (35-point window), panels D–F to normalization, panels G–I to second derivative (35-point window) combined with normalization, and panels J–L to second derivative (35-point window) combined with smoothing (35-point window). Note: r Cal=1.00 represents a rounded value; the actual value was slightly below 1.00.

A

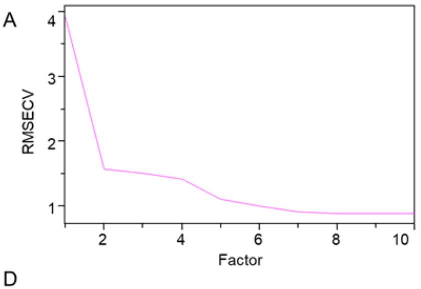


B

| | Variance Percent | Cumulative | RMSEC | RMSECV | r Cal |
|---|---|---|---|---|---|
| Factor1 | 82.47 | 82.47 | 3.85 | 3.92 | 0.86 |
| Factor2 | 12.64 | 95.12 | 1.51 | 1.57 | 0.98 |
| Factor3 | 1.00 | 96.12 | 1.43 | 1.51 | 0.98 |
| Factor4 | 2.91 | 99.03 | 1.31 | 1.42 | 0.99 |
| Factor5 | 0.37 | 99.40 | 0.88 | 1.11 | 0.99 |
| Factor6 | 0.09 | 99.49 | 0.80 | 1.00 | 1.00 |
| Factor7 | 0.23 | 99.72 | 0.71 | 0.92 | 1.00 |
| Factor8 | 0.05 | 99.77 | 0.65 | 0.89 | 1.00 |
| Factor9 | 0.03 | 99.81 | 0.57 | 0.89 | 1.00 |
| Factor10 | 0.03 | 99.84 | 0.52 | 0.89 | 1.00 |

C

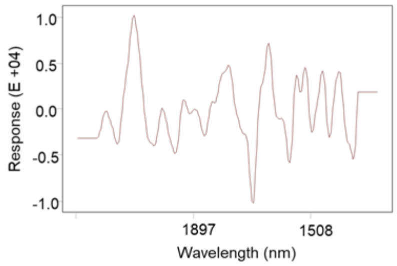


D

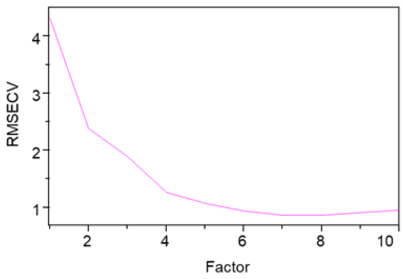


E

| | Variance Percent | Cumulative | RMSEC | RMSECV | r Cal |
|---|---|---|---|---|---|
| Factor1 | 97.07 | 97.07 | 4.25 | 4.31 | 0.83 |
| Factor2 | 2.24 | 99.31 | 2.30 | 2.38 | 0.95 |
| Factor3 | 0.16 | 99.47 | 1.74 | 1.90 | 0.97 |
| Factor4 | 0.32 | 99.79 | 1.15 | 1.27 | 0.99 |
| Factor5 | 0.04 | 99.83 | 0.95 | 1.08 | 0.99 |
| Factor6 | 0.04 | 99.87 | 0.82 | 0.95 | 0.99 |
| Factor7 | 0.08 | 99.95 | 0.69 | 0.88 | 1.00 |
| Factor8 | 0.01 | 99.96 | 0.62 | 0.87 | 1.00 |
| Factor9 | 0.01 | 99.97 | 0.56 | 0.92 | 1.00 |
| Factor10 | 0.00 | 99.98 | 0.45 | 0.96 | 1.00 |

F

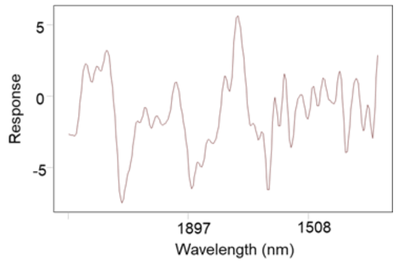


G

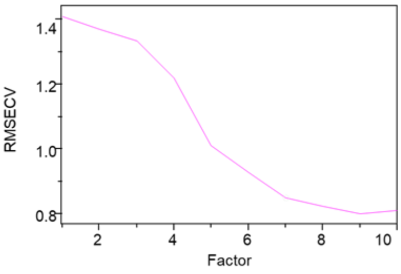


H

| | Variance Percent | Cumulative | RMSEC | RMSECV | r Cal |
|---|---|---|---|---|---|
| Factor1 | 81.64 | 81.64 | 1.38 | 1.41 | 0.98 |
| Factor2 | 8.93 | 90.57 | 1.33 | 1.37 | 0.99 |
| Factor3 | 6.07 | 96.64 | 1.27 | 1.33 | 0.99 |
| Factor4 | 1.13 | 97.76 | 0.92 | 1.22 | 0.99 |
| Factor5 | 0.40 | 98.17 | 0.79 | 1.01 | 1.00 |
| Factor6 | 0.80 | 98.97 | 0.70 | 0.93 | 1.00 |
| Factor7 | 0.15 | 99.11 | 0.63 | 0.85 | 1.00 |
| Factor8 | 0.11 | 99.22 | 0.52 | 0.82 | 1.00 |
| Factor9 | 0.11 | 99.34 | 0.50 | 0.80 | 1.00 |
| Factor10 | 0.16 | 99.49 | 0.46 | 0.81 | 1.00 |

I

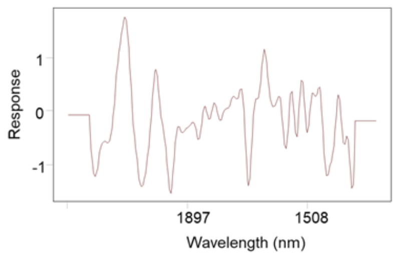


J

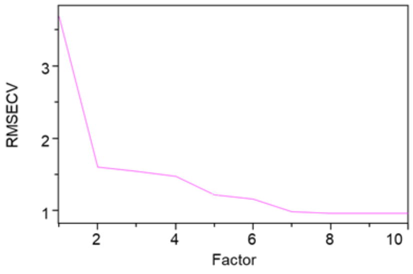


K

| | Variance Percent | Cumulative | RMSEC | RMSECV | r Cal |
|---|---|---|---|---|---|
| Factor1 | 82.01 | 82.01 | 3.62 | 3.69 | 0.88 |
| Factor2 | 13.29 | 95.30 | 1.54 | 1.61 | 0.98 |
| Factor3 | 0.67 | 95.97 | 1.43 | 1.55 | 0.98 |
| Factor4 | 3.02 | 98.99 | 1.37 | 1.48 | 0.98 |
| Factor5 | 0.40 | 99.39 | 0.97 | 1.23 | 0.99 |
| Factor6 | 0.22 | 99.60 | 0.93 | 1.17 | 0.99 |
| Factor7 | 0.19 | 99.80 | 0.82 | 1.00 | 0.99 |
| Factor8 | 0.04 | 99.83 | 0.79 | 0.97 | 1.00 |
| Factor9 | 0.03 | 99.86 | 0.74 | 0.97 | 1.00 |
| Factor10 | 0.02 | 99.88 | 0.72 | 0.97 | 1.00 |

L

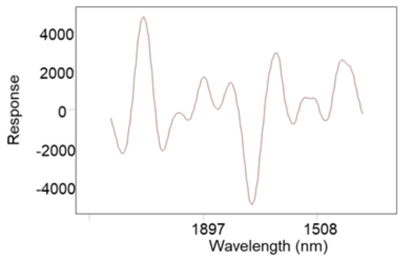

Figure S4. Selection of the optimal number of latent variables by leave-one-out cross-validation (LOOCV) and corresponding regression vectors for the wheat PLSR models using different spectral preprocessing strategies, each applied after mean-centering. For each preprocessing strategy, the three panels from left to right present the RMSECV plot against the number of latent variables (factors), the corresponding explained variance and statistical performance, and the regression vector, respectively. The optimal number of latent variables is highlighted in gray. Panels A–C correspond to second derivative preprocessing (35-point window), panels D–F to normalization, panels G–I to second derivative (35-point window) combined with normalization, and panels J–L to second derivative (35-point window) combined with smoothing (35-point window). Note: r Cal=1.00 represents a rounded value; the actual value was slightly below 1.00.

A

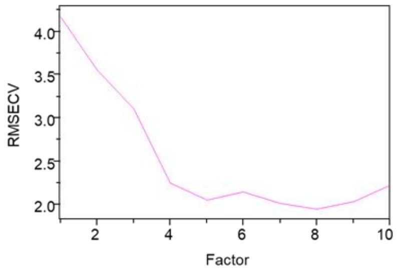


B

| | Variance Percent | Cumulative | RMSEC | RMSECV | r Cal |
|---|---|---|---|---|---|
| Factor1 | 70.96 | 70.96 | 4.08 | 4.17 | 0.79 |
| Factor2 | 15.51 | 86.47 | 3.41 | 3.56 | 0.86 |
| Factor3 | 8.74 | 95.21 | 2.90 | 3.10 | 0.91 |
| Factor4 | 2.21 | 97.42 | 1.99 | 2.25 | 0.96 |
| Factor5 | 1.16 | 98.58 | 1.85 | 2.05 | 0.96 |
| Factor6 | 0.24 | 98.83 | 1.71 | 2.15 | 0.97 |
| Factor7 | 0.63 | 99.45 | 1.43 | 2.01 | 0.98 |
| Factor8 | 0.11 | 99.56 | 1.37 | 1.94 | 0.98 |
| Factor9 | 0.06 | 99.63 | 1.21 | 2.04 | 0.99 |
| Factor10 | 0.05 | 99.67 | 1.14 | 2.22 | 0.99 |

C

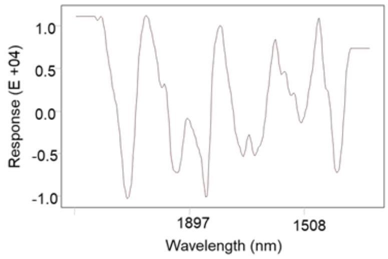


D

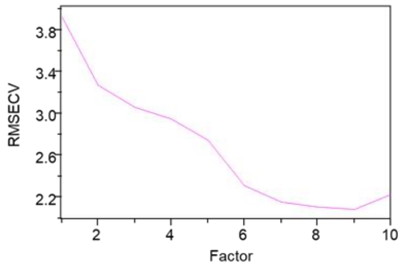


E

| | Variance Percent | Cumulative | RMSEC | RMSECV | r Cal |
|---|---|---|---|---|---|
| Factor1 | 91.46 | 91.46 | 3.84 | 3.92 | 0.82 |
| Factor2 | 2.65 | 94.11 | 3.01 | 3.27 | 0.90 |
| Factor3 | 2.81 | 96.92 | 2.84 | 3.06 | 0.91 |
| Factor4 | 0.93 | 97.85 | 2.68 | 2.95 | 0.92 |
| Factor5 | 0.95 | 98.81 | 2.33 | 2.74 | 0.94 |
| Factor6 | 0.91 | 99.71 | 1.92 | 2.31 | 0.96 |
| Factor7 | 0.06 | 99.77 | 1.64 | 2.16 | 0.97 |
| Factor8 | 0.06 | 99.83 | 1.40 | 2.10 | 0.98 |
| Factor9 | 0.04 | 99.87 | 1.21 | 2.08 | 0.99 |
| Factor10 | 0.01 | 99.88 | 1.03 | 2.23 | 0.99 |

F

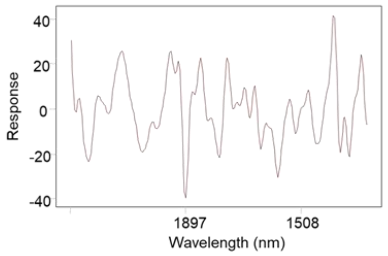


G

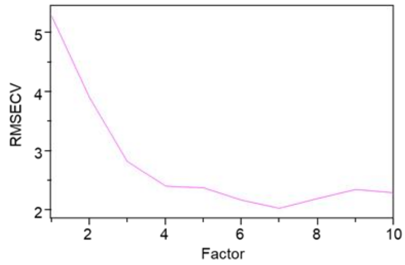


H

| | Variance Percent | Cumulative | RMSEC | RMSECV | r Cal |
|---|---|---|---|---|---|
| Factor1 | 48.87 | 48.87 | 5.12 | 5.29 | 0.64 |
| Factor2 | 28.72 | 77.59 | 3.66 | 3.91 | 0.84 |
| Factor3 | 12.33 | 89.92 | 2.60 | 2.83 | 0.92 |
| Factor4 | 4.00 | 93.92 | 2.19 | 2.41 | 0.95 |
| Factor5 | 2.06 | 95.98 | 2.11 | 2.38 | 0.95 |
| Factor6 | 2.02 | 97.99 | 1.57 | 2.17 | 0.98 |
| Factor7 | 0.12 | 98.11 | 1.45 | 2.02 | 0.98 |
| Factor8 | 0.42 | 98.53 | 1.35 | 2.19 | 0.98 |
| Factor9 | 0.25 | 98.78 | 1.23 | 2.35 | 0.99 |
| Factor10 | 0.25 | 99.03 | 1.06 | 2.29 | 0.99 |

I

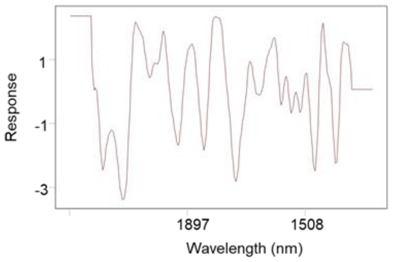


J

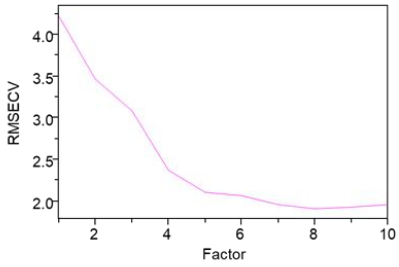


K

| | Variance Percent | Cumulative | RMSEC | RMSECV | r Cal |
|---|---|---|---|---|---|
| Factor1 | 67.42 | 67.42 | 4.12 | 4.22 | 0.79 |
| Factor2 | 18.26 | 85.67 | 3.30 | 3.47 | 0.87 |
| Factor3 | 8.43 | 94.10 | 2.88 | 3.08 | 0.91 |
| Factor4 | 3.48 | 97.58 | 2.15 | 2.37 | 0.95 |
| Factor5 | 1.27 | 98.85 | 1.92 | 2.10 | 0.96 |
| Factor6 | 0.44 | 99.29 | 1.84 | 2.07 | 0.97 |
| Factor7 | 0.32 | 99.61 | 1.54 | 1.96 | 0.98 |
| Factor8 | 0.14 | 99.74 | 1.50 | 1.91 | 0.98 |
| Factor9 | 0.07 | 99.81 | 1.41 | 1.93 | 0.98 |
| Factor10 | 0.02 | 99.83 | 1.40 | 1.96 | 0.98 |

L

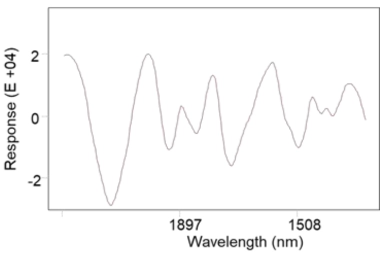

Figure S5. Selection of the optimal number of latent variables by leave-one-out cross-validation (LOOCV) and corresponding regression vectors for the sesame PLSR models using different spectral preprocessing strategies, each applied after mean-centering. For each preprocessing strategy, the three panels from left to right present the RMSECV plot against the number of latent variables (factors), the corresponding explained variance and statistical performance, and the regression vector, respectively. The optimal number of latent variables is highlighted in gray. Panels A–C correspond to second derivative preprocessing (35-point window), panels D–F to normalization, panels G–I to second derivative (35-point window) combined with normalization, and panels J–L to second derivative (35-point window) combined with smoothing (35-point window). Note: r Cal=1.00 represents a rounded value; the actual value was slightly below 1.00.

A

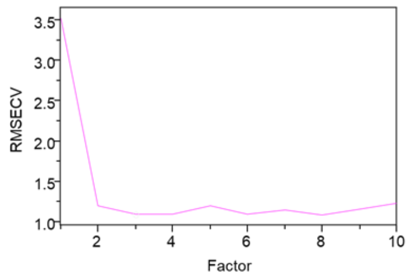


B

| | Variance Percent | Cumulative | RMSEC | RMSECV | r Cal |
|---|---|---|---|---|---|
| Factor1 | 91.44 | 91.44 | 3.46 | 3.52 | 0.88 |
| Factor2 | 3.22 | 94.66 | 1.11 | 1.20 | 0.99 |
| Factor3 | 1.57 | 96.22 | 1.06 | 1.09 | 0.99 |
| Factor4 | 3.16 | 99.38 | 1.03 | 1.10 | 0.99 |
| Factor5 | 0.20 | 99.58 | 0.94 | 1.19 | 0.99 |
| Factor6 | 0.21 | 99.78 | 0.90 | 1.10 | 0.99 |
| Factor7 | 0.07 | 99.86 | 0.77 | 1.14 | 0.99 |
| Factor8 | 0.01 | 99.87 | 0.71 | 1.08 | 1.00 |
| Factor9 | 0.02 | 99.88 | 0.67 | 1.16 | 1.00 |
| Factor10 | 0.02 | 99.90 | 0.58 | 1.23 | 1.00 |

C

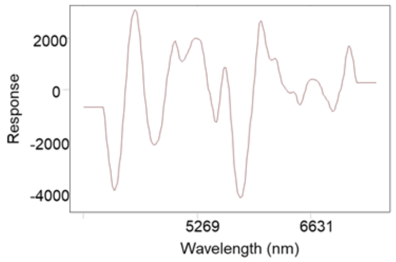


D

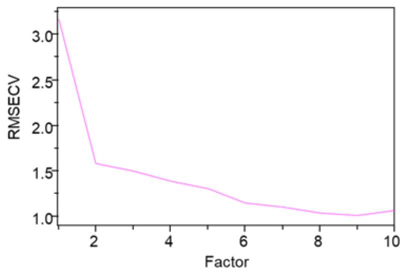


E

| | Variance Percent | Cumulative | RMSEC | RMSECV | r Cal |
|---|---|---|---|---|---|
| Factor1 | 98.68 | 98.68 | 3.13 | 3.17 | 0.90 |
| Factor2 | 0.64 | 99.32 | 1.50 | 1.58 | 0.98 |
| Factor3 | 0.20 | 99.52 | 1.40 | 1.50 | 0.98 |
| Factor4 | 0.29 | 99.81 | 1.25 | 1.39 | 0.99 |
| Factor5 | 0.10 | 99.91 | 1.17 | 1.31 | 0.99 |
| Factor6 | 0.06 | 99.96 | 0.97 | 1.14 | 0.99 |
| Factor7 | 0.01 | 99.97 | 0.80 | 1.11 | 0.99 |
| Factor8 | 0.01 | 99.98 | 0.60 | 1.04 | 1.00 |
| Factor9 | 0.00 | 99.98 | 0.54 | 1.01 | 1.00 |
| Factor10 | 0.00 | 99.98 | 0.47 | 1.07 | 1.00 |

F

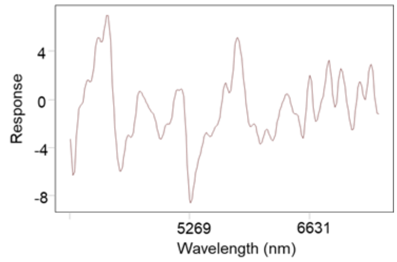


G

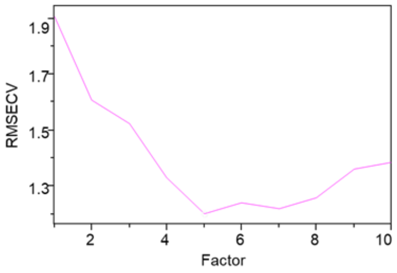


H

| | Variance Percent | Cumulative | RMSEC | RMSECV | r Cal |
|---|---|---|---|---|---|
| Factor1 | 54.78 | 54.78 | 1.80 | 1.91 | 0.97 |
| Factor2 | 26.58 | 81.35 | 1.54 | 1.61 | 0.98 |
| Factor3 | 11.76 | 93.12 | 1.46 | 1.52 | 0.98 |
| Factor4 | 3.53 | 96.64 | 1.11 | 1.33 | 0.99 |
| Factor5 | 1.80 | 98.44 | 1.04 | 1.20 | 0.99 |
| Factor6 | 0.41 | 98.85 | 0.88 | 1.24 | 0.99 |
| Factor7 | 0.14 | 98.99 | 0.82 | 1.22 | 0.99 |
| Factor8 | 0.11 | 99.10 | 0.76 | 1.26 | 1.00 |
| Factor9 | 0.08 | 99.19 | 0.66 | 1.36 | 1.00 |
| Factor10 | 0.08 | 99.26 | 0.62 | 1.39 | 1.00 |

I

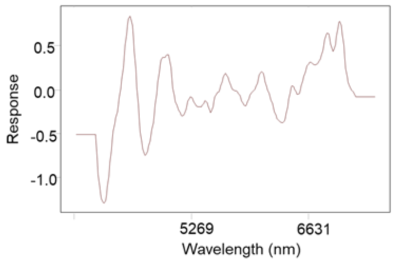


J

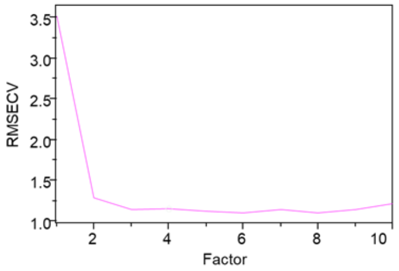


K

| | Variance Percent | Cumulative | RMSEC | RMSECV | r Cal |
|---|---|---|---|---|---|
| Factor1 | 90.74 | 90.74 | 3.45 | 3.52 | 0.88 |
| Factor2 | 3.79 | 94.54 | 1.20 | 1.29 | 0.99 |
| Factor3 | 0.96 | 95.50 | 1.05 | 1.14 | 0.99 |
| Factor4 | 0.79 | 96.29 | 1.05 | 1.15 | 0.99 |
| Factor5 | 3.27 | 99.56 | 1.00 | 1.12 | 0.99 |
| Factor6 | 0.29 | 99.85 | 0.95 | 1.10 | 0.99 |
| Factor7 | 0.04 | 99.89 | 0.87 | 1.14 | 0.99 |
| Factor8 | 0.01 | 99.90 | 0.83 | 1.11 | 0.99 |
| Factor9 | 0.03 | 99.93 | 0.83 | 1.14 | 0.99 |
| Factor10 | 0.01 | 99.93 | 0.81 | 1.21 | 0.99 |

L

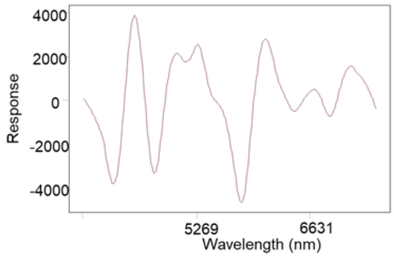

Figure S6. Selection of the optimal number of latent variables by leave-10-out cross-validation and corresponding regression vectors for the soybean PLSR models using different spectral preprocessing strategies, each applied after mean-centering. For each preprocessing strategy, the three panels from left to right present the RMSECV plot against the number of latent variables (factors), the corresponding explained variance and statistical performance, and the regression vector, respectively. The optimal number of latent variables is highlighted in gray. Panels A–C correspond to second derivative preprocessing (35 points), panels D–F to normalization, panels G–I to second derivative combined with normalization (35 points), and panels J–L to second derivative combined with smoothing (35 points). Note: r Cal=1.00 represents a rounded value; the actual value was slightly below 1.00.

A

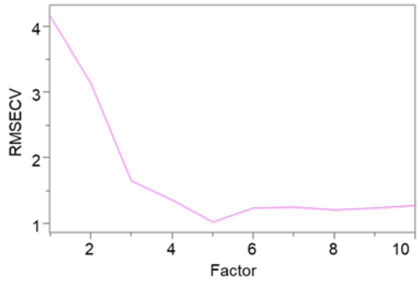


B

| | Variance Percent | Cumulative | RMSEC | RMSECV | r Cal |
|---|---|---|---|---|---|
| Factor1 | 75.24 | 75.24 | 4.19 | 4.15 | 0.82 |
| Factor2 | 10.79 | 86.03 | 3.02 | 3.13 | 0.91 |
| Factor3 | 4.92 | 90.95 | 1.35 | 1.65 | 0.98 |
| Factor4 | 3.63 | 94.58 | 1.26 | 1.36 | 0.99 |
| Factor5 | 4.92 | 99.50 | 0.95 | 1.02 | 0.99 |
| Factor6 | 0.03 | 99.54 | 0.87 | 1.24 | 0.99 |
| Factor7 | 0.11 | 99.64 | 0.81 | 1.25 | 0.99 |
| Factor8 | 0.06 | 99.71 | 0.77 | 1.22 | 1.00 |
| Factor9 | 0.05 | 99.75 | 0.69 | 1.24 | 1.00 |
| Factor10 | 0.03 | 99.78 | 0.61 | 1.28 | 1.00 |

C

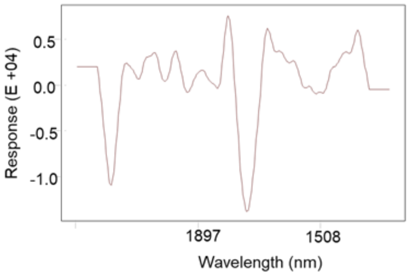


D

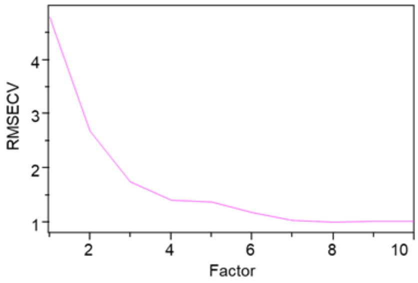


E

| | Variance Percent | Cumulative | RMSEC | RMSECV | r Cal |
|---|---|---|---|---|---|
| Factor1 | 97.24 | 97.24 | 4.83 | 4.80 | 0.75 |
| Factor2 | 0.95 | 98.20 | 2.50 | 2.68 | 0.94 |
| Factor3 | 0.93 | 99.13 | 1.57 | 1.75 | 0.98 |
| Factor4 | 0.23 | 99.36 | 1.31 | 1.41 | 0.98 |
| Factor5 | 0.41 | 99.77 | 1.21 | 1.37 | 0.99 |
| Factor6 | 0.09 | 99.86 | 0.96 | 1.18 | 0.99 |
| Factor7 | 0.01 | 99.87 | 0.88 | 1.03 | 0.99 |
| Factor8 | 0.09 | 99.97 | 0.76 | 0.99 | 1.00 |
| Factor9 | 0.01 | 99.97 | 0.66 | 1.02 | 1.00 |
| Factor10 | 0.00 | 99.98 | 0.53 | 1.01 | 1.00 |

F

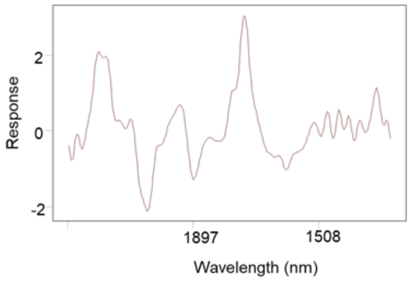


G

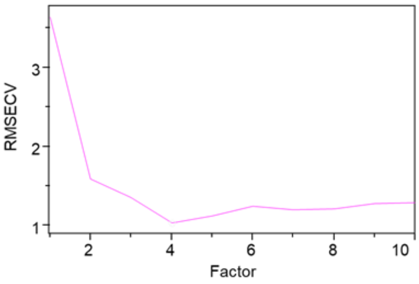


H

| | Variance Percent | Cumulative | RMSEC | RMSECV | r Cal |
|---|---|---|---|---|---|
| Factor1 | 46.39 | 46.39 | 3.61 | 3.64 | 0.87 |
| Factor2 | 20.57 | 66.96 | 1.41 | 1.59 | 0.98 |
| Factor3 | 5.05 | 72.01 | 1.18 | 1.36 | 0.99 |
| Factor4 | 25.88 | 97.88 | 0.95 | 1.03 | 0.99 |
| Factor5 | 0.29 | 98.17 | 0.90 | 1.12 | 0.99 |
| Factor6 | 0.50 | 98.67 | 0.81 | 1.24 | 0.99 |
| Factor7 | 0.26 | 98.93 | 0.77 | 1.20 | 1.00 |
| Factor8 | 0.11 | 99.04 | 0.69 | 1.21 | 1.00 |
| Factor9 | 0.14 | 99.18 | 0.64 | 1.27 | 1.00 |
| Factor10 | 0.12 | 99.30 | 0.58 | 1.28 | 1.00 |

I

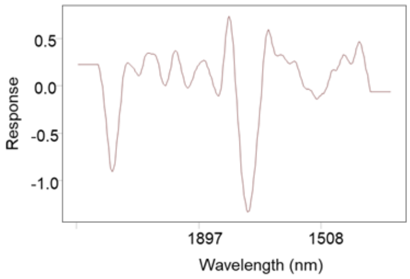


J

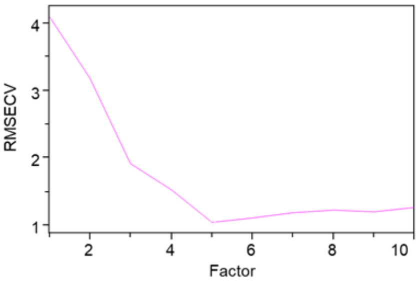


K

| | Variance Percent | Cumulative | RMSEC | RMSECV | r Cal |
|---|---|---|---|---|---|
| Factor1 | 70.46 | 70.46 | 4.13 | 4.09 | 0.82 |
| Factor2 | 14.00 | 84.46 | 3.09 | 3.17 | 0.91 |
| Factor3 | 6.01 | 90.47 | 1.57 | 1.91 | 0.98 |
| Factor4 | 6.62 | 97.08 | 1.39 | 1.52 | 0.98 |
| Factor5 | 2.50 | 99.59 | 0.97 | 1.04 | 0.99 |
| Factor6 | 0.09 | 99.67 | 0.94 | 1.11 | 0.99 |
| Factor7 | 0.09 | 99.76 | 0.92 | 1.19 | 0.99 |
| Factor8 | 0.08 | 99.84 | 0.89 | 1.23 | 0.99 |
| Factor9 | 0.02 | 99.87 | 0.86 | 1.19 | 0.99 |
| Factor10 | 0.02 | 99.89 | 0.82 | 1.26 | 0.99 |

L

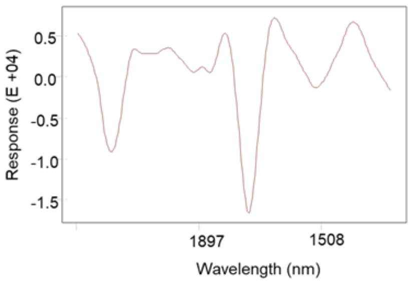

Figure S7. Selection of the optimal number of latent variables by leave-10-out cross-validation and corresponding regression vectors for the peanut PLSR models using different spectral preprocessing strategies, each applied after mean-centering. For each preprocessing strategy, the three panels from left to right present the RMSECV plot against the number of latent variables (factors), the corresponding explained variance and statistical performance, and the regression vector, respectively. The optimal number of latent variables is highlighted in gray. Panels A–C correspond to second derivative preprocessing (35 points), panels D–F to normalization, panels G–I to second derivative combined with normalization (35 points), and panels J–L to second derivative combined with smoothing (35 points). Note: r Cal=1.00 represents a rounded value; the actual value was slightly below 1.00.

A

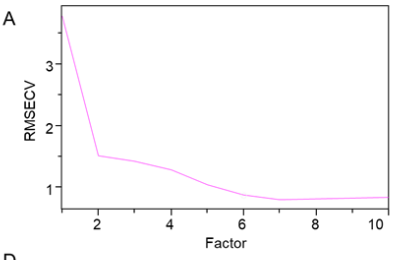


B

| | Variance Percent | Cumulative | RMSEC | RMSECV | r Cal |
|---|---|---|---|---|---|
| Factor1 | 82.47 | 82.47 | 3.85 | 3.78 | 0.86 |
| Factor2 | 12.64 | 95.12 | 1.51 | 1.51 | 0.98 |
| Factor3 | 1.00 | 96.12 | 1.43 | 1.43 | 0.98 |
| Factor4 | 2.91 | 99.03 | 1.31 | 1.28 | 0.99 |
| Factor5 | 0.37 | 99.40 | 0.88 | 1.04 | 0.99 |
| Factor6 | 0.09 | 99.49 | 0.80 | 0.88 | 1.00 |
| Factor7 | 0.23 | 99.72 | 0.71 | 0.79 | 1.00 |
| Factor8 | 0.05 | 99.77 | 0.65 | 0.81 | 1.00 |
| Factor9 | 0.03 | 99.81 | 0.57 | 0.83 | 1.00 |
| Factor10 | 0.03 | 99.84 | 0.52 | 0.84 | 1.00 |

C

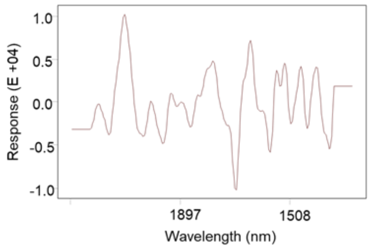


D

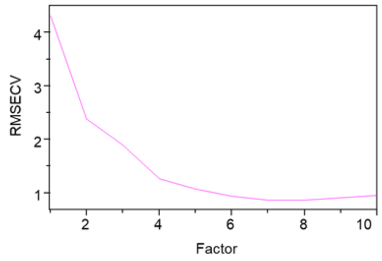


E

| | Variance Percent | Cumulative | RMSEC | RMSECV | r Cal |
|---|---|---|---|---|---|
| Factor1 | 97.07 | 97.07 | 4.25 | 4.16 | 0.83 |
| Factor2 | 2.24 | 99.31 | 2.30 | 2.30 | 0.95 |
| Factor3 | 0.16 | 99.47 | 1.74 | 1.77 | 0.97 |
| Factor4 | 0.32 | 99.79 | 1.15 | 1.25 | 0.99 |
| Factor5 | 0.04 | 99.83 | 0.95 | 1.11 | 0.99 |
| Factor6 | 0.04 | 99.87 | 0.82 | 0.95 | 0.99 |
| Factor7 | 0.08 | 99.95 | 0.69 | 0.84 | 1.00 |
| Factor8 | 0.01 | 99.96 | 0.62 | 0.82 | 1.00 |
| Factor9 | 0.01 | 99.97 | 0.56 | 0.86 | 1.00 |
| Factor10 | 0.00 | 99.98 | 0.45 | 0.87 | 1.00 |

F

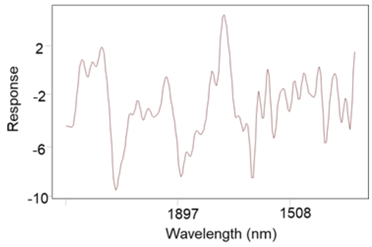


G

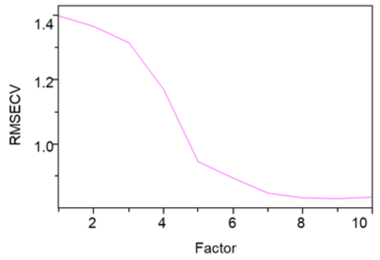


H

| | Variance Percent | Cumulative | RMSEC | RMSECV | r Cal |
|---|---|---|---|---|---|
| Factor1 | 81.64 | 81.64 | 1.38 | 1.40 | 0.98 |
| Factor2 | 8.93 | 90.57 | 1.33 | 1.37 | 0.99 |
| Factor3 | 6.07 | 96.64 | 1.27 | 1.31 | 0.99 |
| Factor4 | 1.13 | 97.76 | 0.92 | 1.17 | 0.99 |
| Factor5 | 0.40 | 98.17 | 0.79 | 0.95 | 1.00 |
| Factor6 | 0.80 | 98.97 | 0.70 | 0.89 | 1.00 |
| Factor7 | 0.15 | 99.11 | 0.63 | 0.85 | 1.00 |
| Factor8 | 0.11 | 99.22 | 0.52 | 0.83 | 1.00 |
| Factor9 | 0.11 | 99.34 | 0.50 | 0.83 | 1.00 |
| Factor10 | 0.16 | 99.49 | 0.46 | 0.83 | 1.00 |

I

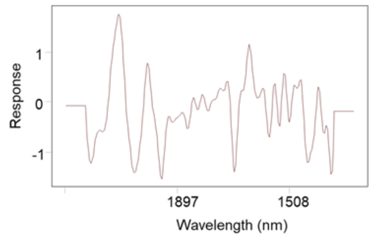


J

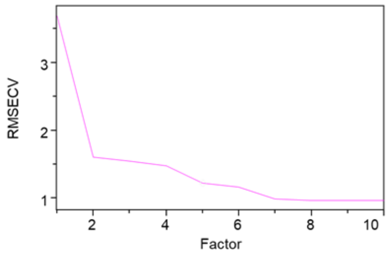


K

| | Variance Percent | Cumulative | RMSEC | RMSECV | r Cal |
|---|---|---|---|---|---|
| Factor1 | 82.01 | 82.01 | 3.62 | 3.55 | 0.88 |
| Factor2 | 13.29 | 95.30 | 1.54 | 1.54 | 0.98 |
| Factor3 | 0.67 | 95.97 | 1.43 | 1.55 | 0.98 |
| Factor4 | 3.02 | 98.99 | 1.37 | 1.40 | 0.98 |
| Factor5 | 0.40 | 99.39 | 0.97 | 1.17 | 0.99 |
| Factor6 | 0.22 | 99.60 | 0.93 | 1.03 | 0.99 |
| Factor7 | 0.19 | 99.80 | 0.82 | 0.90 | 0.99 |
| Factor8 | 0.04 | 99.83 | 0.79 | 0.90 | 1.00 |
| Factor9 | 0.03 | 99.86 | 0.74 | 0.97 | 1.00 |
| Factor10 | 0.02 | 99.88 | 0.72 | 1.00 | 1.00 |

L

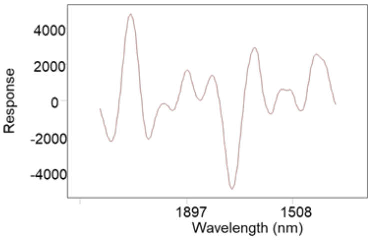

Figure S8. Selection of the optimal number of latent variables by leave-10-out cross-validation and corresponding regression vectors for the wheat PLSR models using different spectral preprocessing strategies, each applied after mean-centering. For each preprocessing strategy, the three panels from left to right present the RMSECV plot against the number of latent variables (factors), the corresponding explained variance and statistical performance, and the regression vector, respectively. The optimal number of latent variables is highlighted in gray. Panels A–C correspond to second derivative preprocessing (35 points), panels D–F to normalization, panels G–I to second derivative combined with normalization (35 points), and panels J–L to second derivative combined with smoothing (35 points). Note: r Cal=1.00 represents a rounded value; the actual value was slightly below 1.00.

A

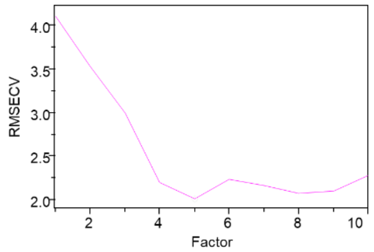


B

| | Variance Percent | Cumulative | RMSEC | RMSECV | r Cal |
|---|---|---|---|---|---|
| Factor1 | 70.96 | 70.96 | 4.08 | 4.11 | 0.79 |
| Factor2 | 15.51 | 86.47 | 3.41 | 3.53 | 0.86 |
| Factor3 | 8.74 | 95.21 | 2.90 | 3.00 | 0.91 |
| Factor4 | 2.21 | 97.42 | 1.99 | 2.20 | 0.96 |
| Factor5 | 1.16 | 98.58 | 1.85 | 2.01 | 0.96 |
| Factor6 | 0.24 | 98.83 | 1.71 | 2.24 | 0.97 |
| Factor7 | 0.63 | 99.45 | 1.43 | 2.16 | 0.98 |
| Factor8 | 0.11 | 99.56 | 1.37 | 2.08 | 0.98 |
| Factor9 | 0.06 | 99.63 | 1.21 | 2.10 | 0.99 |
| Factor10 | 0.05 | 99.67 | 1.14 | 2.28 | 0.99 |

C

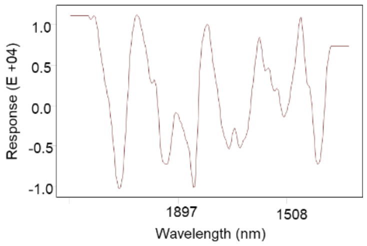


D

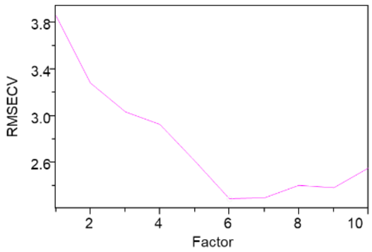


E

| | Variance Percent | Cumulative | RMSEC | RMSECV | r Cal |
|---|---|---|---|---|---|
| Factor1 | 91.46 | 91.46 | 3.84 | 3.86 | 0.82 |
| Factor2 | 2.65 | 94.11 | 3.01 | 3.28 | 0.90 |
| Factor3 | 2.81 | 96.92 | 2.84 | 3.03 | 0.91 |
| Factor4 | 0.93 | 97.85 | 2.68 | 2.93 | 0.92 |
| Factor5 | 0.95 | 98.81 | 2.33 | 2.61 | 0.94 |
| Factor6 | 0.91 | 99.71 | 1.92 | 2.29 | 0.96 |
| Factor7 | 0.06 | 99.77 | 1.64 | 2.30 | 0.97 |
| Factor8 | 0.06 | 99.83 | 1.40 | 2.40 | 0.98 |
| Factor9 | 0.04 | 99.87 | 1.21 | 2.38 | 0.99 |
| Factor10 | 0.01 | 99.88 | 1.03 | 2.55 | 0.99 |

F

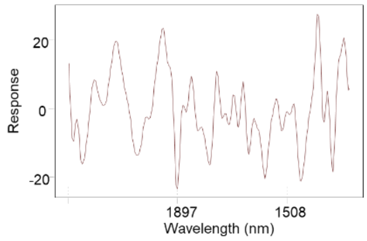


G

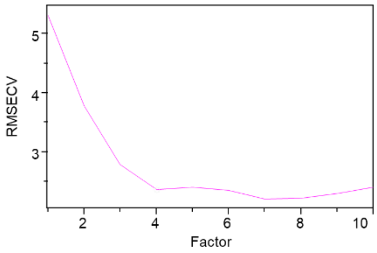


H

| | Variance Percent | Cumulative | RMSEC | RMSECV | r Cal |
|---|---|---|---|---|---|
| Factor1 | 48.87 | 48.87 | 5.12 | 5.31 | 0.64 |
| Factor2 | 28.72 | 77.59 | 3.66 | 3.78 | 0.84 |
| Factor3 | 12.33 | 89.92 | 2.60 | 2.80 | 0.92 |
| Factor4 | 4.00 | 93.92 | 2.19 | 2.37 | 0.95 |
| Factor5 | 2.06 | 95.98 | 2.11 | 2.41 | 0.95 |
| Factor6 | 2.02 | 97.99 | 1.57 | 2.35 | 0.98 |
| Factor7 | 0.12 | 98.11 | 1.45 | 2.20 | 0.98 |
| Factor8 | 0.42 | 98.53 | 1.35 | 2.22 | 0.98 |
| Factor9 | 0.25 | 98.78 | 1.23 | 2.30 | 0.99 |
| Factor10 | 0.25 | 99.03 | 1.06 | 2.41 | 0.99 |

I

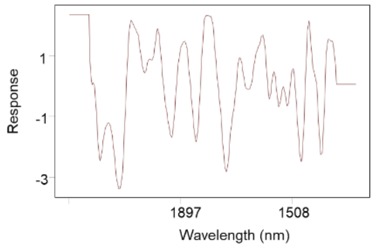


J

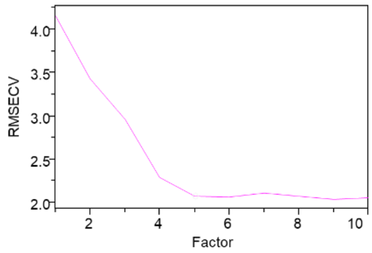


K

| | Variance Percent | Cumulative | RMSEC | RMSECV | r Cal |
|---|---|---|---|---|---|
| Factor1 | 67.42 | 67.42 | 4.12 | 4.16 | 0.79 |
| Factor2 | 18.26 | 85.67 | 3.30 | 3.43 | 0.87 |
| Factor3 | 8.43 | 94.10 | 2.88 | 2.96 | 0.91 |
| Factor4 | 3.48 | 97.58 | 2.15 | 2.29 | 0.95 |
| Factor5 | 1.27 | 98.85 | 1.92 | 2.07 | 0.96 |
| Factor6 | 0.44 | 99.29 | 1.84 | 2.06 | 0.97 |
| Factor7 | 0.32 | 99.61 | 1.54 | 2.11 | 0.98 |
| Factor8 | 0.14 | 99.74 | 1.50 | 2.07 | 0.98 |
| Factor9 | 0.07 | 99.81 | 1.41 | 2.03 | 0.98 |
| Factor10 | 0.02 | 99.83 | 1.40 | 2.05 | 0.98 |

L

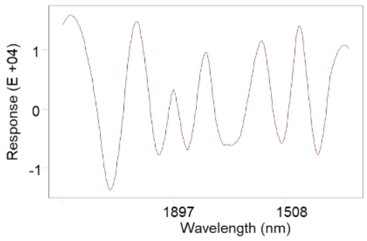

Figure S9. Selection of the optimal number of latent variables by leave-10-out cross-validation and corresponding regression vectors for the sesame PLSR models using different spectral preprocessing strategies, each applied after mean-centering. For each preprocessing strategy, the three panels from left to right present the RMSECV plot against the number of latent variables (factors), the corresponding explained variance and statistical performance, and the regression vector, respectively. The optimal number of latent variables is highlighted in gray. Panels A–C correspond to second derivative preprocessing (35 points), panels D–F to normalization, panels G–I to second derivative combined with normalization (35 points), and panels J–L to second derivative combined with smoothing (35 points). Note: r Cal=1.00 represents a rounded value; the actual value was slightly below 1.00.

A

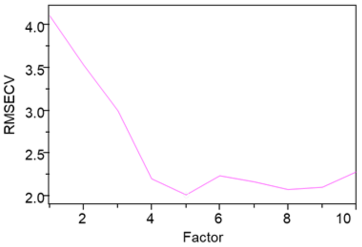


B

| | Variance Percent | Cumulative | RMSEC | RMSECV | r Cal |
|---|---|---|---|---|---|
| Factor1 | 70.96 | 70.96 | 4.08 | 4.11 | 0.79 |
| Factor2 | 15.51 | 86.47 | 3.41 | 3.53 | 0.86 |
| Factor3 | 8.74 | 95.21 | 2.90 | 3.00 | 0.91 |
| Factor4 | 2.21 | 97.42 | 1.99 | 2.20 | 0.96 |
| Factor5 | 1.16 | 98.58 | 1.85 | 2.01 | 0.96 |
| Factor6 | 0.24 | 98.83 | 1.71 | 2.24 | 0.97 |
| Factor7 | 0.63 | 99.45 | 1.43 | 2.16 | 0.98 |
| Factor8 | 0.11 | 99.56 | 1.37 | 2.08 | 0.98 |
| Factor9 | 0.06 | 99.63 | 1.21 | 2.10 | 0.99 |
| Factor10 | 0.05 | 99.67 | 1.14 | 2.28 | 0.99 |

C

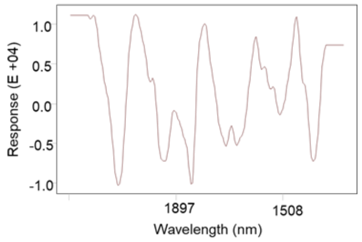


D

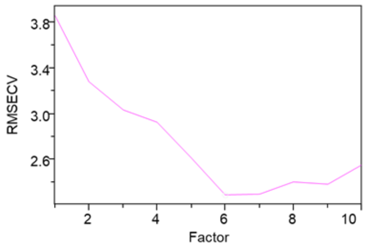


E

| | Variance Percent | Cumulative | RMSEC | RMSECV | r Cal |
|---|---|---|---|---|---|
| Factor1 | 91.46 | 91.46 | 3.84 | 3.86 | 0.82 |
| Factor2 | 2.65 | 94.11 | 3.01 | 3.28 | 0.90 |
| Factor3 | 2.81 | 96.92 | 2.84 | 3.03 | 0.91 |
| Factor4 | 0.93 | 97.85 | 2.68 | 2.93 | 0.92 |
| Factor5 | 0.95 | 98.81 | 2.33 | 2.61 | 0.94 |
| Factor6 | 0.91 | 99.71 | 1.92 | 2.29 | 0.96 |
| Factor7 | 0.06 | 99.77 | 1.64 | 2.30 | 0.97 |
| Factor8 | 0.06 | 99.83 | 1.40 | 2.40 | 0.98 |
| Factor9 | 0.04 | 99.87 | 1.21 | 2.38 | 0.99 |
| Factor10 | 0.01 | 99.88 | 1.03 | 2.55 | 0.99 |

F

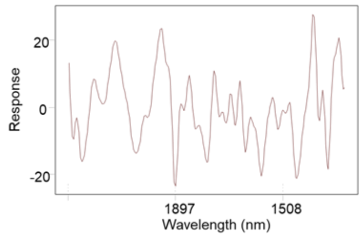


G

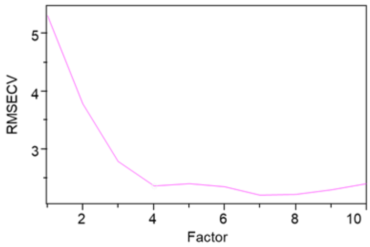


H

| | Variance Percent | Cumulative | RMSEC | RMSECV | r Cal |
|---|---|---|---|---|---|
| Factor1 | 48.87 | 48.87 | 5.12 | 5.31 | 0.64 |
| Factor2 | 28.72 | 77.59 | 3.66 | 3.78 | 0.84 |
| Factor3 | 12.33 | 89.92 | 2.60 | 2.80 | 0.92 |
| Factor4 | 4.00 | 93.92 | 2.19 | 2.37 | 0.95 |
| Factor5 | 2.06 | 95.98 | 2.11 | 2.41 | 0.95 |
| Factor6 | 2.02 | 97.99 | 1.57 | 2.35 | 0.98 |
| Factor7 | 0.12 | 98.11 | 1.45 | 2.20 | 0.98 |
| Factor8 | 0.42 | 98.53 | 1.35 | 2.22 | 0.98 |
| Factor9 | 0.25 | 98.78 | 1.23 | 2.30 | 0.99 |
| Factor10 | 0.25 | 99.03 | 1.06 | 2.41 | 0.99 |

I

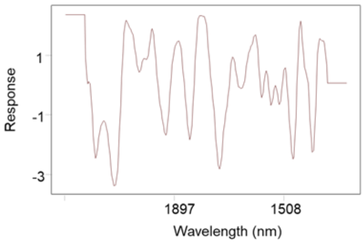


J

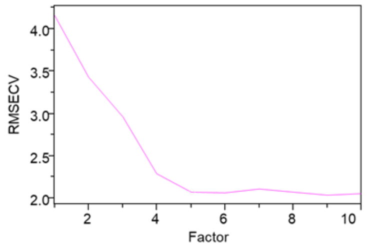


K

| | Variance Percent | Cumulative | RMSEC | RMSECV | r Cal |
|---|---|---|---|---|---|
| Factor1 | 67.42 | 67.42 | 4.12 | 4.16 | 0.79 |
| Factor2 | 18.26 | 85.67 | 3.30 | 3.43 | 0.87 |
| Factor3 | 8.43 | 94.10 | 2.88 | 2.96 | 0.91 |
| Factor4 | 3.48 | 97.58 | 2.15 | 2.29 | 0.95 |
| Factor5 | 1.27 | 98.85 | 1.92 | 2.07 | 0.96 |
| Factor6 | 0.44 | 99.29 | 1.84 | 2.06 | 0.97 |
| Factor7 | 0.32 | 99.61 | 1.54 | 2.11 | 0.98 |
| Factor8 | 0.14 | 99.74 | 1.50 | 2.07 | 0.98 |
| Factor9 | 0.07 | 99.81 | 1.41 | 2.03 | 0.98 |
| Factor10 | 0.02 | 99.83 | 1.40 | 2.05 | 0.98 |

L

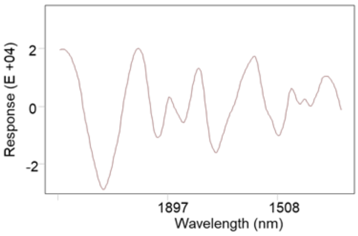

Supplement: Supplementary file 1 [file foods-15-03434-s001.zip › foods-4432904-supplementary.pdf]
